# Dynamics of Bcl-xL in Water and Membrane: Molecular Simulations

**DOI:** 10.1371/journal.pone.0076837

**Published:** 2013-10-08

**Authors:** Atanu Maity, Seema Yadav, Chandra S. Verma, Shubhra Ghosh Dastidar

**Affiliations:** 1 Bioinformatics Centre, Bose Institute, Kolkata, West Bengal, India; 2 Bioinformatics Institute, Agency for Science, Technology and Research, Singapore, Singapore; 3 Department of Biological Sciences, National University of Singapore, Singapore, Singapore; 4 School of Biological Sciences, Nanyang Technological University, Singapore, Singapore; King's College, London, United Kingdom

## Abstract

The Bcl2 family of proteins is capable of switching the apoptotic machinery by directly controlling the release of apoptotic factors from the mitochondrial outer membrane. They have ‘pro’ and ‘anti’-apoptotic subgroups of proteins which antagonize each other’s function; however a detailed atomistic understanding of their mechanisms based on the dynamical events, particularly in the membrane, is lacking. Using molecular dynamics simulations totaling 1.6µs we outline the major differences between the conformational dynamics in water and in membrane. Using implicit models of solvent and membrane, the simulated results reveal a picture that is in agreement with the ‘hit-and run’ concept which states that BH3-only peptides displace the tail (which acts as a pseudo substrate of the protein itself) from its binding pocket; this helps the membrane association of the protein after which the BH3 peptide becomes free. From simulations, Bcl-xL appears to be auto-inhibited by its C-terminal tail that embeds into and covers the hydrophobic binding pocket. However the tail is unable to energetically compete with BH3-peptides in water. In contrast, in the membrane, neither the tail nor the BH3-peptides are stable in the binding pocket and appear to be easily dissociated off as the pocket expands in response to the hydrophobic environment. This renders the binding pocket large and open, thus receptive to interactions with other protein partners. Principal components of the motions are dramatically different in the aqueous and in the membrane environments and provide clues regarding the conformational transitions that Bcl-xL undergoes in the membrane, in agreement with the biochemical data.

## Introduction

The apoptotic machinery of cells acts to prevent disease. It is normally kept at very low levels [1] but gets activated upon irreparable damage or under the threat of a disease. Cancerous cells adopt mechanisms to bypass ‘apoptotic’ activation to ensure their survival [2]. This is often executed by over-expression of factors that suppress the functions of apoptosis inducers. For example, the Bcl2 family of proteins, whose nomenclature ‘Bcl2’ comes from one of its members, has pro- (e.g. Bak, Bax, etc.) and anti- (e.g. Bcl-xl, Bcl2, Bcl-w etc.) apoptotic subgroups which antagonize each other under conditions that ensure normal cell cycling [3]. They are known to be active at the outer membrane of the mitocondria. Pro-apoptotic members are capable of self-organizing into oligomers that make the membrane porous. These pores are sufficiently large to allow the passage of apoptotic factors from the space between the mitocondrial inner and outer membranes [4]. This is a key step for triggering the apoptosis. The role of the anti apoptotic members is to prevent this oligomerization in the membrane (i.e. pore formation) by forming heterodimeric complexes with the pro-apoptotic members, thereby neutralizing their action. This interplay ensures a suitable balance between the pro- and anti-apoptotic proteins in the cytosol and within the membrane, thus ensuring normal cell cycle. Depending upon the need, this balance shifts. Abnormalities in the balance between these two groups and over-activation of anti-apoptotic members can cause cancer by ensuring the survival of the diseased cell. A promising strategy to combat the disease is to administer inhibitors that would selectively block the anti apoptotic members of the Bcl2 family [5-8]. Recently there have been some clinical successes through the development of small molecules such as ABT737, ABT263, TW-37 etc. [9-11]

Bcl2 family members share four homology domains (termed as BH1, BH2, BH3 & BH4) although not every member necessarily contains all the domains, e.g. the BH3-only proteins contain only one domain [12]. All these proteins are largely helical as it has been seen in X-ray-crystallographic [13-15] and NMR-derived structures [16-18]. These proteins are known to be biologically active in the mitochondrial outer membrane [19,20] and there are evidences that they are associate conformational changes when they oligomerize with pro or anti-apoptotic proteins; however structural details are missing [19]. Denisov et al. have hypothesized a ‘hit-and-run’ process [21], whereby Bcl-w, which is a homolog of Bcl-xl, is inhibited by its own C-terminal tail, and this tail is displaced by a BH3-only protein. The free tail anchors in the membrane followed by a large conformational change in the protein which releases the BH3 protein and prepares for pro/anti heterodimer formation. The experimentally determined structure of Bcl-w (PDB ID 1O0L) [22] shows that the tail is bound in a pseudo substrate like fashion. The same is true for Bax whose tail is also bound to its hydrophobic binding pocket (PDB ID 1F16 [23]). This is hyopthesized to be true for its homologues (e.g. Bcl-xl) as well but their structures without ligand have not yet been reported. To examine these processes in atomistic detail, we carry out microsecond molecular dynamics (MD) simulations of Bcl-xl in water and in membrane environments. These studies reveal, for the first time, that significant differences charactertize the conformational ensemble and dynamics of the protein in water and in the membrane, correlating with their active and inactive states.

## Methods

### Modeling of the systems for simulations

**Figure 1:**
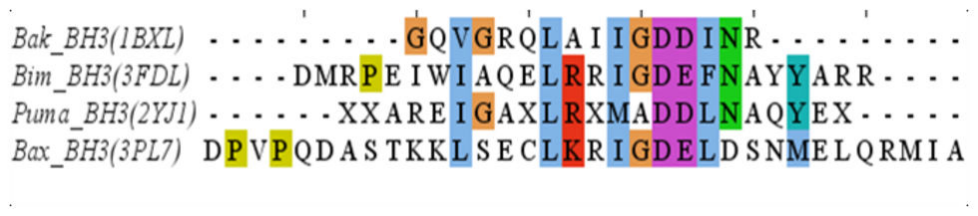
Alignment of BH3^Bak^ (human) with other BH3 only proteins Bim (Bcl2-interacting mediator of cell death), Puma (p53 upregulated modulator of apoptosis), Bax (Bcl-2-associated X protein) of human that are found to bind Bcl-xl in the available structure (PDB ID given in parentheses).

**Figure 2:**
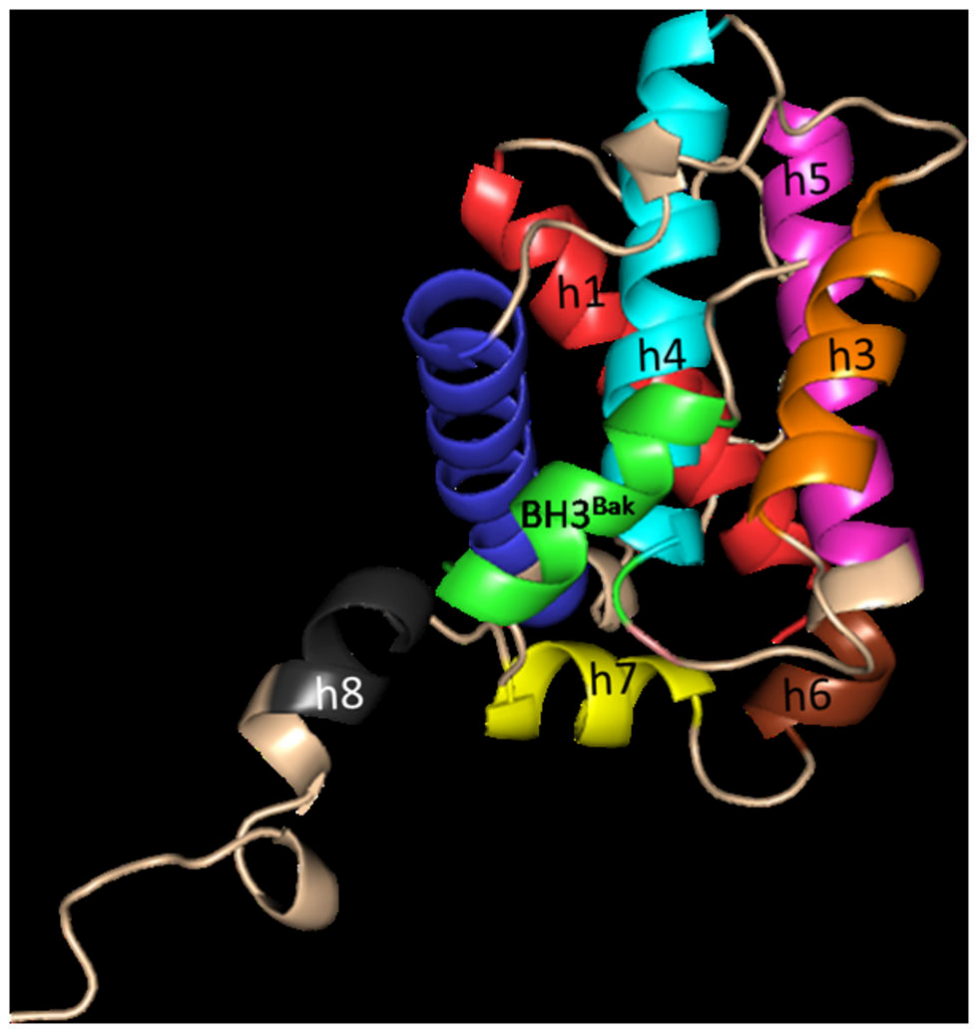
NMR structure of Bcl-xl (residue 1-217, ∆45-84) in complex with Bak BH3 domain(residue 572-587) from PDB ID ‘1BXL’. Different helices of Bcl-xl (h1-h8) are shown in different colors. BH3^Bak^ is shown in green.

**Figure 3:**
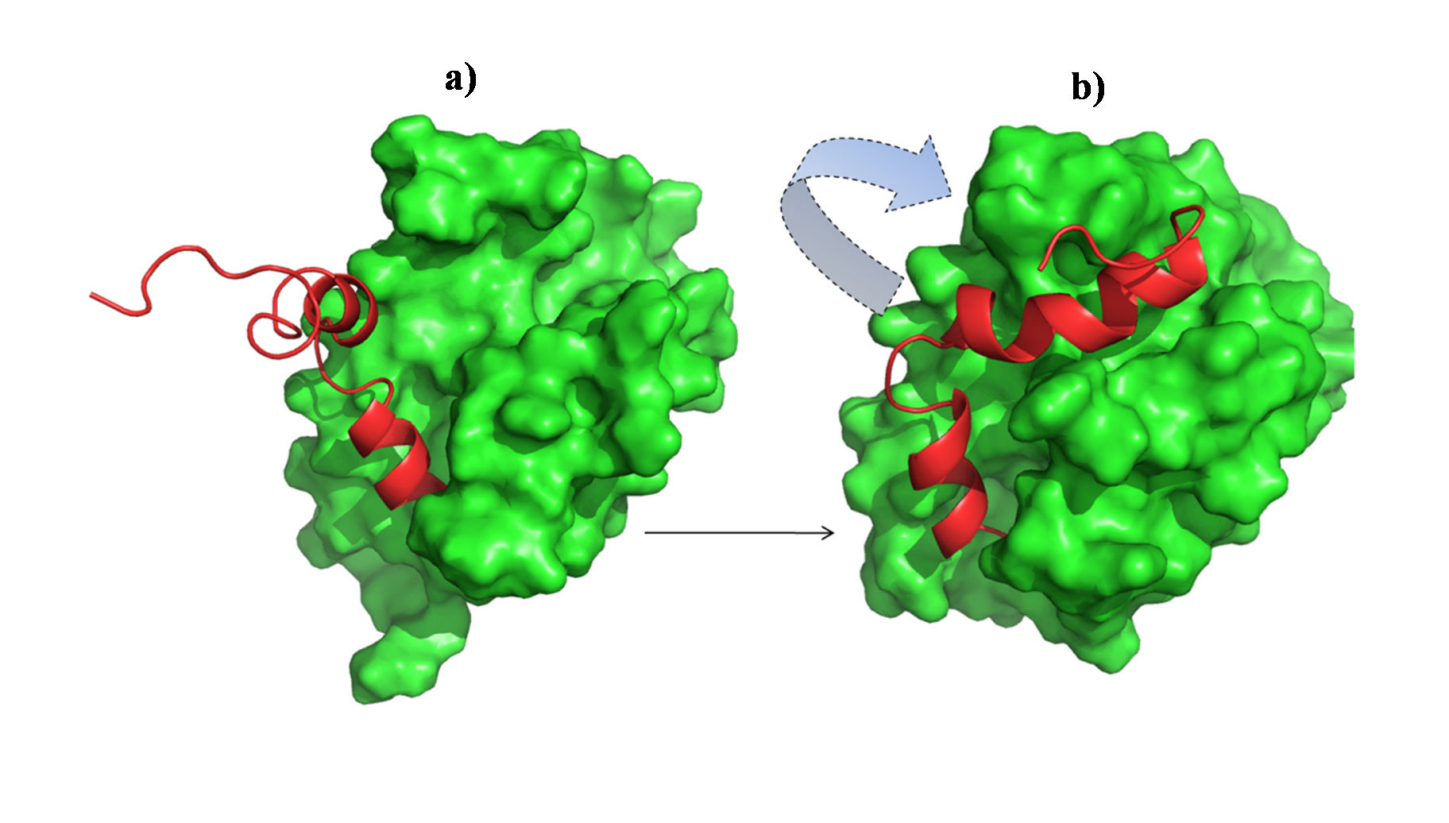
Structure of Bcl-xl in water. (left) at the initial stage and (right) after 60 ns of simulation. Residues 1-192 are in green surface and residues 193-217 are in red ribbon.

The structural coordinates of Bcl-xl complexed with a fragment (BH3 domain) of Bak was obtained from protein databank (PDB ID 1BXL [16]). 1BXL is the NMR-derived structure of anti-apoptotic human Bcl-xl (residues 1-217, excluding residues 45-84, i.e. ∆45-84) complexed with a 16 residue fragment from the pro-apoptotic BH3 domain (residue index in PDB is 572-587) of the Bak protein [24]. Instead of an ensemble (which is usually provided for NMR derived structures) of structures, Sattler et al. have provided an averaged, minimized structure to the protein data bank (PDB) [16]. The BH3 fragment of Bak (BH3^Bak^) shares a close homology with other BH3-only peptides (Figure 1). Bcl-xl consists of five major helices [h1(residues 3-20), h2(residues 85-104), h3(residues 120-130), h4(residues 139-156), h5(residues 160-176)] and three minor helices [h6(residue s179-182), h7(residues 187-194), h8(residues 199-205)] packed together, while Bak is largely helical (see Figure 2). The C-terminal domain (residues 197-217) of Bcl-xl points away from the rest of the protein. The structures of receptor and ligand were capped at their terminal ends (amidated and acetylated at their N- and C-terminals respectively). To mimic the intermolecular binding of the tail to Bcl-xl a portion of the tail (residues 197-217) was docked into the binding pocket of uncomplexed Bcl-xl (residues 1-217, ∆45-84). For this docking the conformation of the tail was extracted from a structure after a 20 ns simulation of uncomplexed Bcl-xl in water (Figure 3). The histidine residues in all the simulations were uniformly kept in a state where the delta nitrogen was protonated (HSD notation in CHARMM format) as predicted by the WHATIF webserver [25]. For a preliminary test with the full length Bak, its crystal structure was taken from PDB (PDB ID 2IMT) [13]. It consists of eight helical (both alpha and 3_10_) regions connected by loops. The central hydrophobic helix (W125-Q144) was surrounded by other amphipathic helices. For docking, PatchDock webserver [26] was used with its default settings. Then the structures were refined using firedock [27]. The system’s energy was minimized well to ensure that they are free from any steric collision prior to setting them for simulations.

### MD Simulations

The systems for simulations were initially prepared using the CHARMM-GUI [28] webserver followed by suitable modifications as required. All the systems were simulated using the CHARMM [29] simulation program, applying the CHARMM22 force field [30] with CMAP corrections [31]. The GBSW model [32] of implicit water and membrane were used to represent the surrounding environment of the proteins. The optimized version of GBSW radii was used as it has been shown to reduce the 'over-stabilization' problem of protein-protein interactions in implicit solvent [33]. GBSW model is a successful implicit model and has been shown to reproduce a number of properties of proteins in membrane and transmembrane domains such as membrane insertion of melittin, a 26-residue alpha helical peptide [34], interfacial folding and membrane insertion of different types of WALP and TMX synthetic peptides [35], binding site dynamics of the ~350-residue protein alpha1A-adrenoceptor antagonist [36]. From the NMR structure (1BXL has only one conformation) the 16-residue BH3^Bak^ peptide was removed from its binding pocket to obtain a structure of Bcl-xl where the binding pocket does not contain any ligand. The uncomplexed and complexed Bcl-xl structures were solvated in implicit water and/or embedded in implicit membrane in separate simulations. The implicit membrane had a 30Å hydrophobic core slab and a 5Å slab on either side to gradually switch the dielectric of the medium from membrane to water. The initial orientation of the protein in the membrane was arbitrary. Altogether 21 trajectories for implicit (including 2-3 independent trajectories for each case, as listed in Table 1) have been run accumulating more than one microsecond (~1.6µs) of sampling. Each structure was energy minimized before carrying out the MD simulations at 300K, using the Langevin dynamics (LD) algorithm. LD used a random force (set by FBETA 5.0 in CHARMM for all heavy atoms) to correspond to collisions with a heat-bath kept at 300K, to ensure a constant temperature of the system. The vibration of the bonds involving hydrogen atoms was frozen using SHAKE [37] which enabled the use of a 2fs integration time step. Snapshots were saved at 2ps intervals. The non-bonded interactions were smoothly switched to zero between 16Å and 20Å. The calculated binding energies have been obtained following the MMGBSW/SA [38] protocol (solute entropies have not been considered). Figures are made using Pymol [39], movies are prepared using VMD [40].

**Table 1:** List of trajectories.

	**System**	**Medium**		**Length of independent trajectories**	**Sampling time**
1	Bcl-xl+BH3^Bak^	Implicit water		100ns, 100ns	200 ns
2	Bcl-xl+BH3^Bak^	Implicit membrane		100ns, 100ns	200 ns
3	Bcl-xl+C-terminal	Implicit water		30ns, 30ns	60ns
4	Uncomplexed Bcl-xl	Implicit water		100 ns, 60ns, 60ns	220 ns
5	Uncomplexed Bcl-xl	Implicit membrane		100ns, 60ns, 60ns	220 ns
6	Uncomplexed BH3^Bak^	Implicit water		100ns, 60ns, 60ns	220 ns
7	Uncomplexed BH3^Bak^	Implicit membrane		100ns, 60ns, 60ns	220 ns
8	Uncomplexed full Bak	Implicit membrane		30ns, 30ns	60ns
9	Bcl-xl+full Bak	Implicit membrane	20ns		20ns
10	Complexed/uncomplexed Bcl-xl	Explicit water/membrane	20 different trajectories of 10ns each		200ns (see Table S6 for details)
			Total		~1.6 µs

For the simulations in explicit solvent and membrane the starting structures were picked up from the different points of the implicit solvent/membrane simulation trajectories randomly. Five extracted structures were used for five independent trajectories for each system i.e. complexed and uncmplexed states in membrane and water; thus total 20 trajectories were run, each of 10ns. The system in water was prepared using TIP3P model [41] of water, ensuring minimum thickness of 9 Å water layer everywhere. To model the protein in membrane, the prequilibrated DOPC(1,2-Dioleoyl-*sn*-glycero-3-phosphocholine) bilayer was obtained from charmm-gui membrane builder [42]. The number of lipid molecules in each leaflet (upper and lower layer) varied from 90 to 110 depending on the conformation of Bcl-xl and its complex with BH3. 25 Å TIP3P water layer was maintained at each side over the lipid headgroups. The CHARMM22 force field with CMAP correction was used for representing the protein, keeping it identical with implcit solvent simulations; whereas lipid parameters were taken from CHARMM27. After heating and equilibration, Langevin Dynamics simulations were carried out at 300K under NPT condition for 10ns length of each trajectory, leading to 200ns altogether. SHAKE [37] algorithm was used to allow 2 fs timestep. Coordinates were saved with an interval of 2 ps for water and 5 ps for membrane. The other details of the explicit water/membrane simulations are provided in the supporting information Text S1. The trajectories are listed in supporting information Table S6.

### Principal Component (PC) Analysis

Principal component analysis (PCA) of an MD trajectory distils out the independent components of concerted motions of atoms that dominate the overall dynamics of the molecule. The mathematical procedures of principal component analysis (PCA) involves an orthogonal transformation to convert a set of observations of possibly correlated variables into a set of linearly uncorrelated variables called principal components. For N atoms, there are 3N-6 degrees of vibrational freedom, thus PCA generates 3N-6 concerted modes or the principal components (PC) of motion. The first few PCs are generally found to capture the essential motions of the system and are associated with the essential dynamics [43,44]. The PCs are obtained mathematically by diagonalizing the variance-covariance matrix (C) of atomic fluctuations:

V^T^CV= A

The diagonal matrix A contains the eigenvalues as the diagonal elements and the V matrix contains the corresponding eigenvectors. The eigenvector with the largest eigenvalue has the highest contribution to the motion i.e. the main structural changes are usually captured along this component, and the other components indexed in the sequence of decreasing eigenvalues have decreasing contributions to the global conformational fluctuations of the molecule. Principal component analysis was performed on the Cα atoms of the residues of the cleft (residues 1-196). The trajectories were projected along the first three PCs for each case and distribution of conformations along the first three principal axes were plotted. Trajectories of fluctuations along different PCs were prepared and have been submitted as movies in the supporting information.

## Results

### Uncomplexed Bcl-*x*l in water

In water, simulations started from uncomplexed Bcl-xl had the hydrophobic binding pocket exposed and its C-terminal tail projected away from the binding pocket (Figure 3a). Within 10ns the partially helical tail folded as it embedded into the binding pocket (see Figure 3b and Movie S1) occluding the latter (Figure S1). This was seen in all the three independent trajectories (Table 1). The ability of the tail to embed into the hydrophobic pocket has also been seen in recent experiments [45] although the mechanism is hypothesized to be intermolecular (arising from a dimer). None of the structural data on the uncomplexed Bcl-xl is available in PDB containing its C-terminal tail. But there are structures of its two homologs Bcl-w and Bax available (PDB IDs 1O0L and 1F16) which show that their C-terminal tails self associate in a pseudo-substrate like fashion; this is consistent with what we see in our simulations.

**Figure 4:**
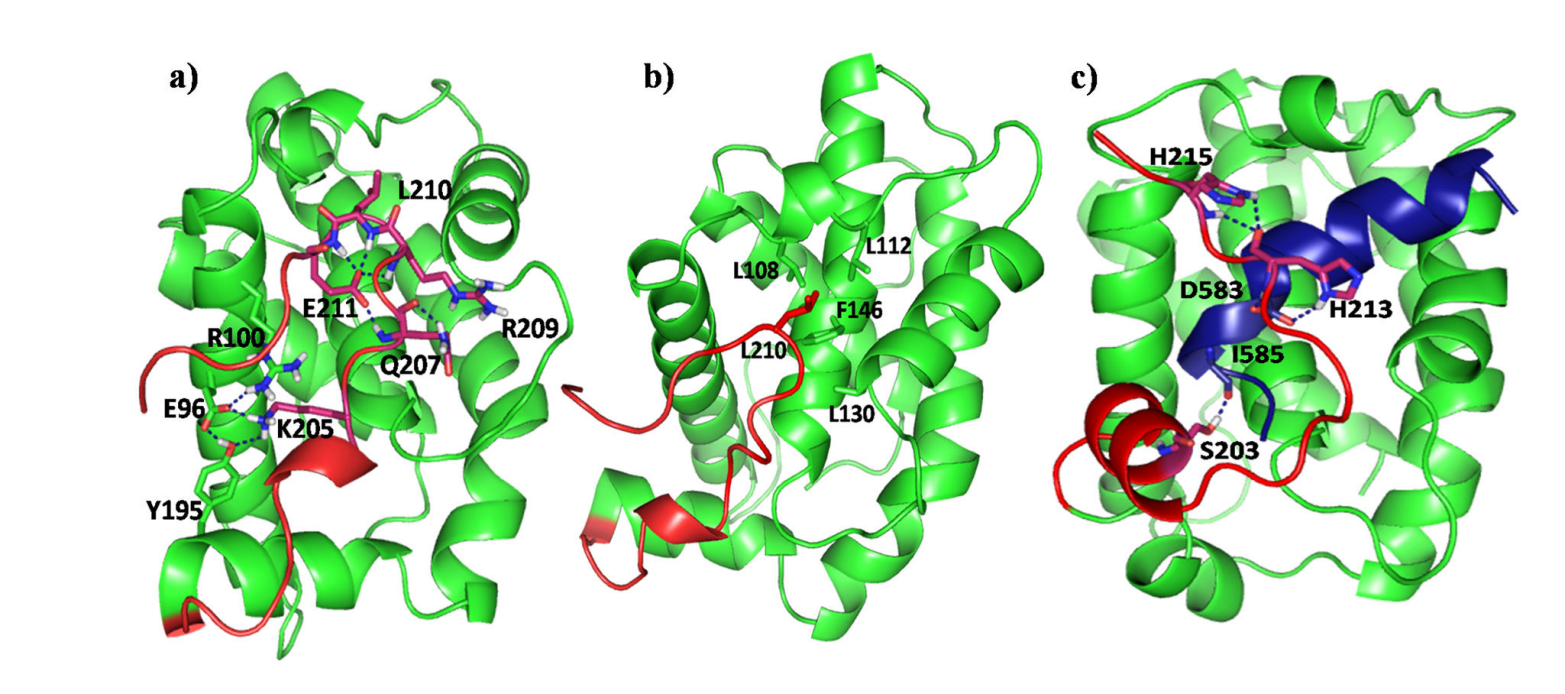
Structure of Bcl-xl in water after 100 ns of simulation. (a) Polar and (b) hydrophobic interactions are shown. Residues forming hydrogen bond are shown in sticks. c) structure of Bcl-xl+BH3^Bak^ complex in water. Residues forming hydrogen bond are shown in sticks, it shows that tail has significant interaction with the BH3 in the complex state, i.e. tail has the tendency to move towards the binding pocket.

Simulations show that the interactions that stabilize the tail in the cleft inlcude salt-bridges between R100, R132, D133 of the cleft and R209, E211 of the tail complemented with hydrophobic interactions of L210 of the tail with L108, L112 and F146 of the cleft (Figure 4a & 4b). Although the tail was initially helical, upon association with the cleft it starts to loose its secondary structure and at around 100ns becomes unstructured but well embedded into the binding groove. It is interesting to note that the intra-helix stabilizations (i.e. intra-helix hydrogen bonds) are replaced by the tail-cleft interactions whose energetic consequence is discussed later. The sequence of the interacting region of the tail is V_192_ELYGNNAAAE _202_SRKGQERLEH_212_ which contains either polar side chains or sidechains with poor hydrophobic anchoring capability (e.g. A,S etc.) except for L194, L210. It interacts with the surface of the protein largely through polar interactions, engaging hydrogen bonds involving (i) E211 sidechain (SC) and backbone of Q207, L210 and E211, (ii) E96 SC and SC of Y195 and K205 (Figure 4a & 4b). The hydrophobic network between F146, L108, V126, L130 of the cleft and L210 of the tail are observed to stabilize the core throughout the trajectory. In the other two independent trajectories, while the orientations of the tail are different, it tends to cover the binding pocket. For example in one trajectory the histidine of the tail is stabilised with the charged side chains of h2 and h3 whereas in the other trajectory, the histidine interacts with residues of h3 and h5 (Figure S2). These kinds of dynamics characterize the interactions of tail regions in other systems also [46]. In summary, while the microstates in the different simulations differ somewhat from each other, it is clear that in aqueous environment, when the ligand (BH3) is absent, the tail acts like a lid which appears to cover the binding pocket through polar and apolar interactions.

### Bcl-*x*l-BH3 complex in water

The BH3 fragment of Bak (sequence G_572_QVGRQLAIIG _582_DDINR) binds to Bcl-xl through a combination of hydrophobic and charged residues that serve to embed it into the Bcl-xl binding pocket(Movie S2). The NMR structure of the complex (1BXL) shows that four residues V574, L578, I581 and I585 of the peptide anchor into the binding pocket of Bcl-xl (residues F97, F105, L108, F146, V126, L130) – a feature that was conserved during the independent simulations also. In addition, residues Q573, R576, D583, D584 and N586 engage in polar interactions with R100, Y101, Q111 and R139. The tail, although not covering the BH3^Bak^ binding groove, makes some stabilizing interactions with the side chains of h2, h3 of Bcl-xl and also with the Bak peptide (Figure 4c); the residues S203, H213, H215, D583^Bak^ and I585^Bak^ form hydrogen bonds. In the other independent trajectory (Table 1), although the location of the tail varies, the overall interactions are very similar. The tail forms a coiled structure and gets stabilized through interactions with h7 (Figure S3) and, in this conformation, the tail is hypothesized to be free (i.e. not complexed into the binding pocket) and thus available for membrane insertion [47]. From the analysis of the NMR structure (1BXL) Sattler et al. [16] suggested the possibility of the formation of several interactions, e.g. E129-R576^Bak^, R139-D583^Bak^ between the Bcl-xl and BH3^Bak^; D583 is highly conserved in the Bcl2 family. These salt-bridge interactions between their sidechains are indeed witnessed in the current simulations, which satisfied our cutoff criteria of 4Å between charged groups’ heavy atoms (Figure S4). R139 is also highly conserved and its mutation affects the complexation with Bax [48], a homolog of Bak; the mutations G138A, R139Q, Y101K and L130A have been reported to inhibit its anti-apoptotic function [16]. The NMR structure of Bcl-xl (1BXL) provides only one conformation; whereas the system is expected to have multiple different conformations in solution due to the possibility of different orientations of the flexible tail which is projected away from the cleft of Bcl-xl. In contrast with 1BXL, the NMR structure of Bcl-w which is a homolog of Bcl-xl, is an ensemble (PDB ID 1ZY3 [21]) of the protein compelxed with BH3^BID^ and the C-terminal tail interacts with the ligand (BH3) in several structures of the ensemble. This is similar to what is seen for the Bcl-xl-BH3 complex in our simulations.

### Uncomplexed Bcl-*x*l in membrane

To simulate the system in a membrane, a reliable starting structure is critical. Since there is no such structural information available in the membrane, the NMR structure was inserted in the membrane to see how the structure evolves in that environment. Until now the actual mechanism of membrane insertion of Bcl2 family members is unclear; the tail is known to help by initiating the process by anchoring into the membrane, followed by insertion of the rest of the protein [19]. Experiments demonstrate [19,48] that proapoptotic proteins get inhibited by anti-apoptotic proteins when they are in the membrane. Hence it is clear that complexation within the membrane is important; the role of the membrane in complexation has been underscored by other groups (Garcia-saez et al. [20]). We now use simulations to examine how the structure may evolve in the membrane to enable complexation.

**Figure 5:**
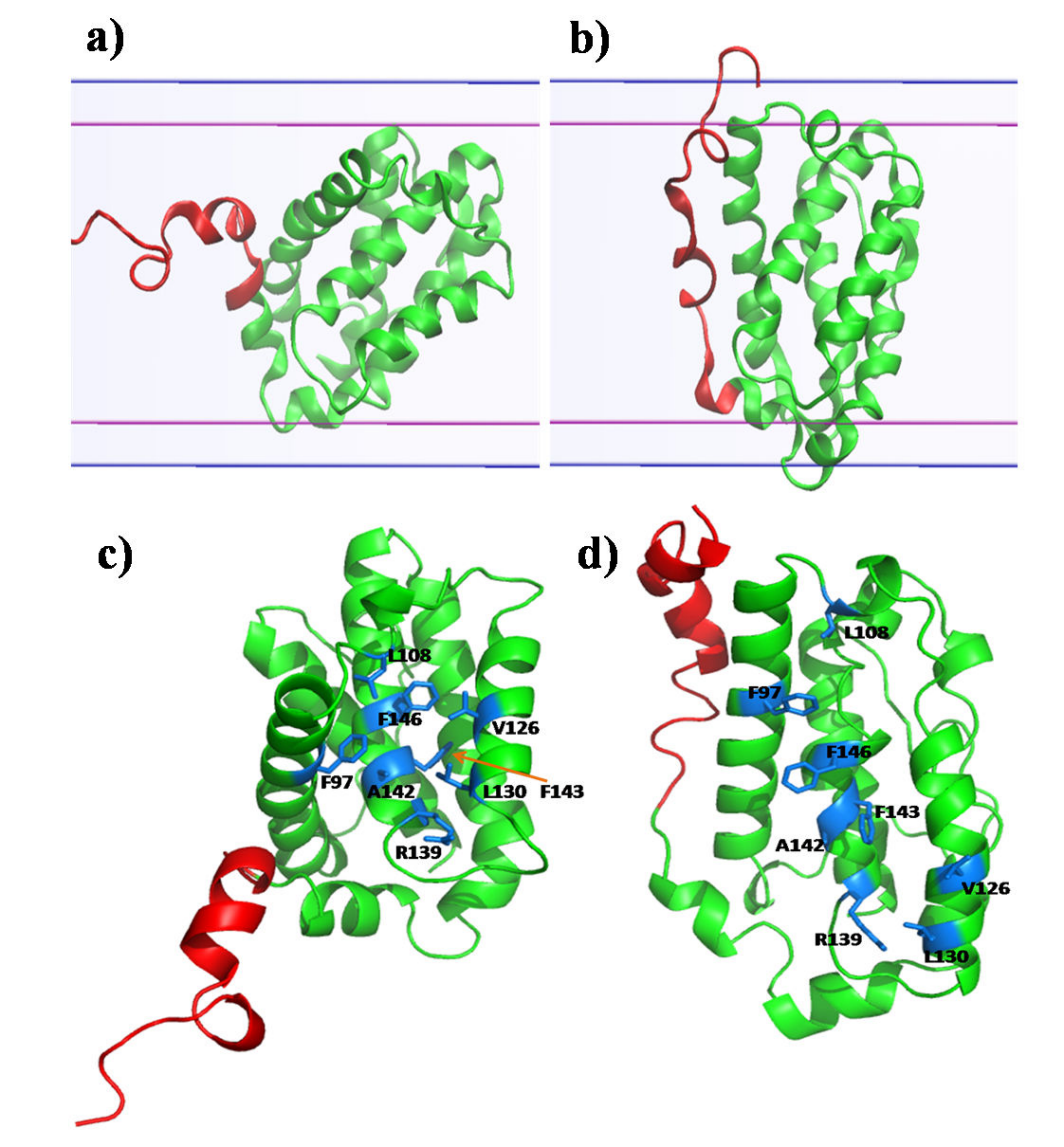
Alignment of helices of Bcl-xl in membrane: snapshots (a) at the very beginning of simulation (b) after 1 ns of simulation. The pairs of straight line indicate the boundary of membrane. Exposure of residues forming the binding pocket in membrane environment. The exposed residues are mostly hydrophobic and shown in cyan stick c) initially and d) after 60 ns of simulation.

**Figure 6:**
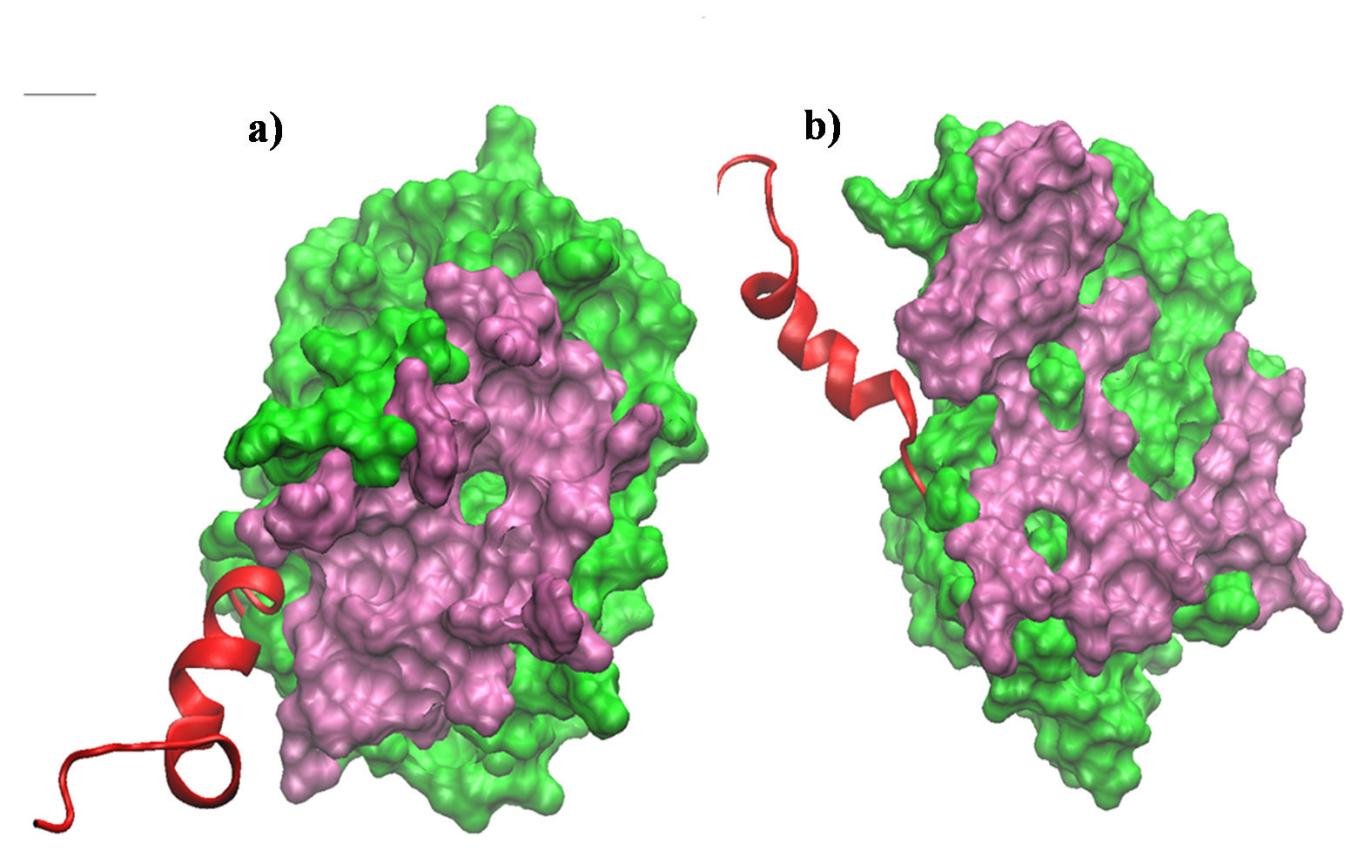
Exposure of the binding pocket, in surface representation. Structures of Bcl-xl without BH3^Bak^ (a) at the starting of the simulation and (b) after 100 ns simulation. The core residues (residue 1 to 196) are shown in green surface and the c-terminal (residue 197 to 217) in red ribbon. The active site residues (which were at a distance 5 Å from the BH3^Bak^ in the NMR structure) are shown in mauve.

In the membrane simulations, irrespective of the arbitrarily chosen starting orientations of Bcl-xl, the protein reorients in such a way that within 3-4 ns, the helices lie along the membrane normal (Figure 5a & 5b and Movie S3). The tail does not bind to the pocket, leaving it open and uncovered (Movie S4). The orientation of the tail alongside helix h2 is initiated by salt bridge interactions between the side chains of h2 (E92, E96, R100) and the residues of the tail (N197, S203, K205). For example the distance between F146 of the protein core and L210 of the tail, initially at ~30Å, stabilizes at ~13Å in water, but never approached closer than ~23Å in the membrane (Figure S5). Within the first few nanoseconds, the protein in the membrane starts to change conformation, exposing its core hydrophobic residues e.g. F143, F146 and A142 (Figure 5c & 5d and RMSD and RGYR plots in Figure S6). The binding pocket flattens (Movie S4), yielding a larger exposed surface which is now in a state that can potentially be stabilized by interactions with a partner with a large surface area around the binding pocket i.e. it can bind a full length protein with a large exposed hydrophobic surface rather than just a peptide (see Figure 6). The flattening of the binding pocket and larger exposure of the hydrophobic residues were observed in all the independent trajectories. Figure S7 shows the solvent accessible surface area of representative snapshots taken at the end of 100ns simulations which clearly shows that in the membrane, the hydrophobic residues get exposed while the polar residues get buried. This now provides a mechanism underlying the experimental observations that suggest oligomerization in the membrane. Throughout all the trajectories, the C-termial tail never showed any tendency to approach the binding pocket. For the other two independent simulations in the membrane, the tail lies along the membrane, leaving the BH3^Bak^ binding pocket unoccupied and differing only in the spatial orientation of the tail residues. For example, E208 is surrounded by C-terminal residues in one trajectory, whereas in the other it is not involved in such interactions; in one trajectory, the tail is on one side of h2, while in the other trajectory it is on the other side (see Figure S8).

### Bcl-*x*l-BH3 complex in membrane

**Figure 7:**
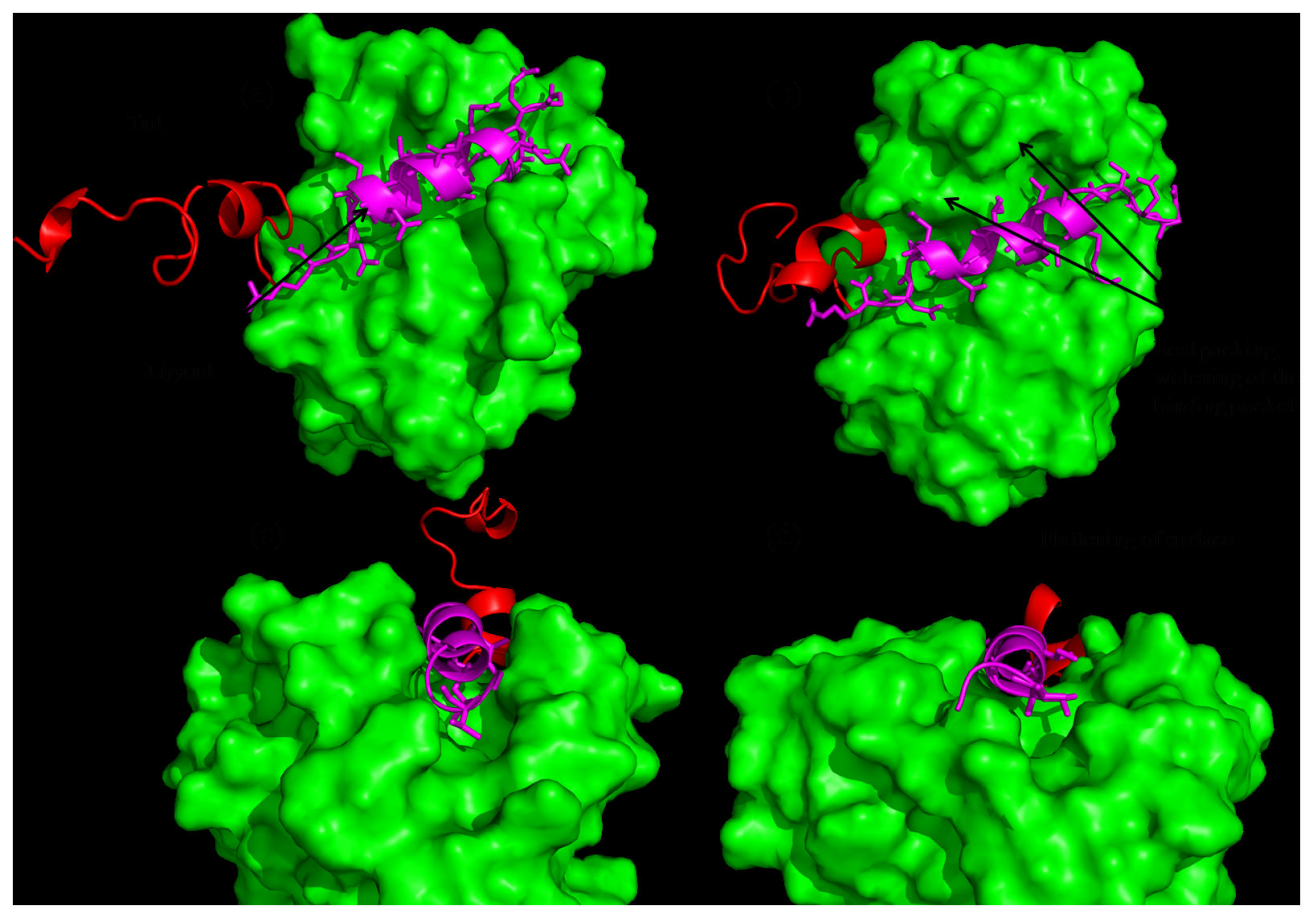
Change of packing at the complex interface in membrane environment as seen from the difference between (a) and (b). The flattening of the binding pocket is shown in (c) and (d).

**Figure 8:**
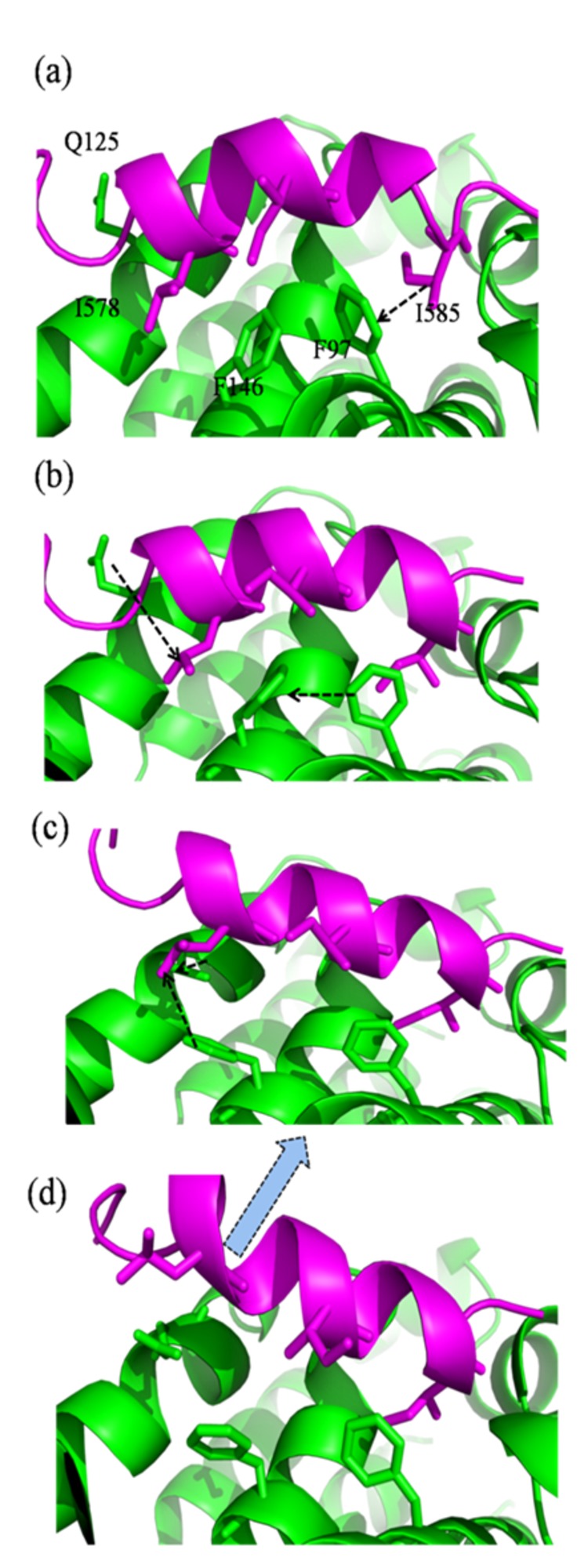
Concerted collisions kicking out of ligand from Bcl-xl-BH3^Bak^ complex in membrane.

When the ligand (BH3^Bak^) is bound to Bcl-xl, it initially forms (Figure S9) electrostatic interactions involving residues D584^Bak^, R204^Bcl-xl^, R139^Bcl-xl,^ D583^Bak^, R576^Bak^, E124^Bcl-xl^,Glu129^Bcl-xl^ and hydrophobic interactions involving residues F105^Bcl-xl^, L578^Bak^, Y101^Bcl-xl^ and F146^Bcl-xl^, L585^Bak^, V141^Bcl-xl^, thus yielding a compact fit. However, we have seen already in the uncomplexed states that the binding pocket starts opening and flattening as the buried hydrophobic residues get exposed to and stabilize in the hydrophobic membrane. This unsuprisingly leads to a weakening of the packing of the ligand (see Figure 7 and Movie S5). A mechanism of the dissociation of the peptide from its bound state in the membrane becomes apparent from the simulations: as shown in Figure 8, I585 of BH3 pushes against F97^Bcl-xl^ that in turns pushes against F146^Bcl-xl^ in a concerted manner (Figure 8(a)-(b)). At the other end of the binding pocket, Q125^Bcl-xl^ pushes against I578^Bak^. It appears that together these concerted collisions result in the N-terminal of the ligand dissociating from its bound state from the cleft (Movie S6). These two events – the collisions coupled to a flattening of the binding pocket are in accord with the ‘hit-and-run’ hypothesis [21] which suggests that the ligand is no longer required in the membrane. Until now it is unclear whether the dissociation of BH3 from Bcl-xl takes place in water or in the membrane. The current study, using MD simulations, suggests that complexation is not favoured in the membrane. This is because the hydrophobic resiudes that normally are sequestered away from water are no more required to be buried as they are stabilized in the hydrophobic environment. So the hydrophobic sidechains at the complex interface lose their packing as mentioned above.

We have mentioned earlier that the protein orients orthogonal to the membrane surface and this probably enables a lateral interaction with partner proteins, thus establishing a mechanism underlying the formation of homo or hetero dimers. What is interesting is that unlike in water, the tail region also remains outside the binding pocket. This is not surprising since the interactions of the tail region are also driven by combinations of hydrophobic and electrostatic interactions whereas the major driving force in the membrane is for the hydrophobic regions of the binding pocket to open up to the hydrophobic membrane. Several changes in the tail region are observed. The tail predominantly has polar residues including charged side chains. In the membrane, since the dielectric constant is low, the polar interactions are strengthened. This arises from the absence of solvation together with a tendency to avoid the hydrophobic membrane, thus resulting in a clustering of the polar regions. Several salt bridge interactions among the polar side chains are observed in this unstructured coil, e.g. Q207^Bcl-xl^ interacts with R587^Bak^ of the ligand, R204^Bcl-xl^ interacts with D584^Bak^ of the ligand and with R100^Bcl-xl^. E96^Bcl-xl^ is surrounded by A200 and A201 with the help of hydrogen bonds of E96 side chain and A200 & A201 backbone. Several contacts are formed by the polar residues of the ligand such as D583^Bak^ and N586^Bak^ with R139^Bcl-xl^. These interactions neutralize each other and thus obtain a stabilizing effect as in the hydrophobic environment of the membrane these charged residues do not experience any other stabilizing interactions, e.g. polar residues get surrounded by polar solvent molecules when exposed in water. Y101 and Q111 make hydrogen bonds with each other and are located near the surface, but at ~48ns, Y101 flips-in towards the binding pocket and no more interacts with Q111. This sequestration of the polar atoms and associated polar interactions, together with the exposure of the hydrophobic residues, appear to contribute to the confomational changes of Bcl-xl in the membrane. The other independent trajectory (Table 1) also shows the same overall features, although some details of the microstates are different. In that trajectory (100 ns), although the concerted collisions were not explicitly observed, nevertheless a weakening of packing at the complex interface was observed (its consequence for the binding energy is discussed later). An important difference here is in the orientation of the tail. In spite of localizing near h2 it adopts an orientation similar to that of the uncomplexed Bcl-xl in the membrane. The orientation was initially guided by salt bridge interactions among residues R204^Bcl-xl^, R100^Bcl-xl^, D584^BH3(bak)^ but by 5 ns, the C-terminal residues get oriented at the edge of the membrane (region with higher polarity) stabilized by electrostatic interactions with R103 (Figure S10).

#### Relative stabilities

**Figure 9:**
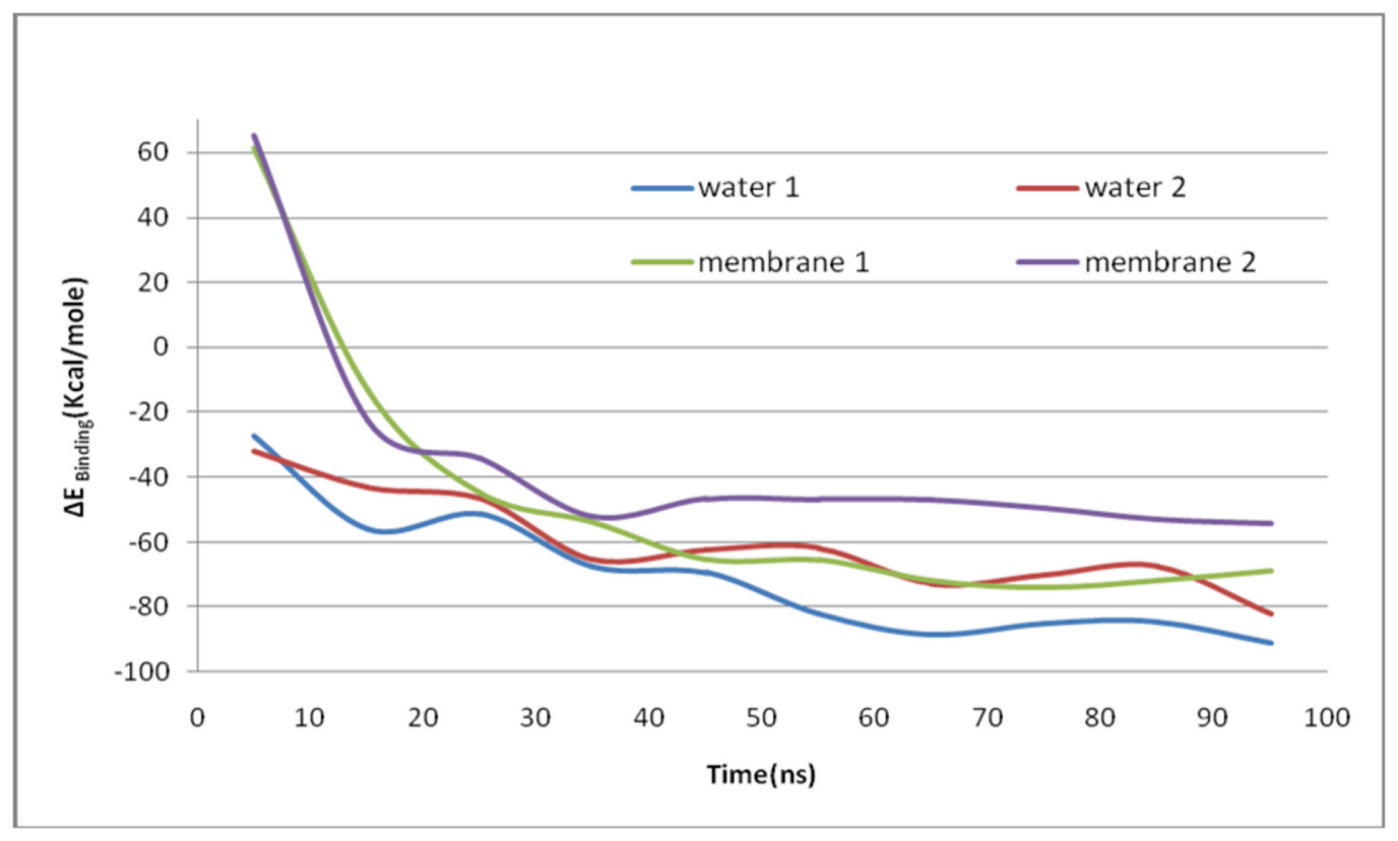
Variation of binding energy of the complex in water and in membrane as a function of time in two independent set of trajectories.

**Figure 10:**
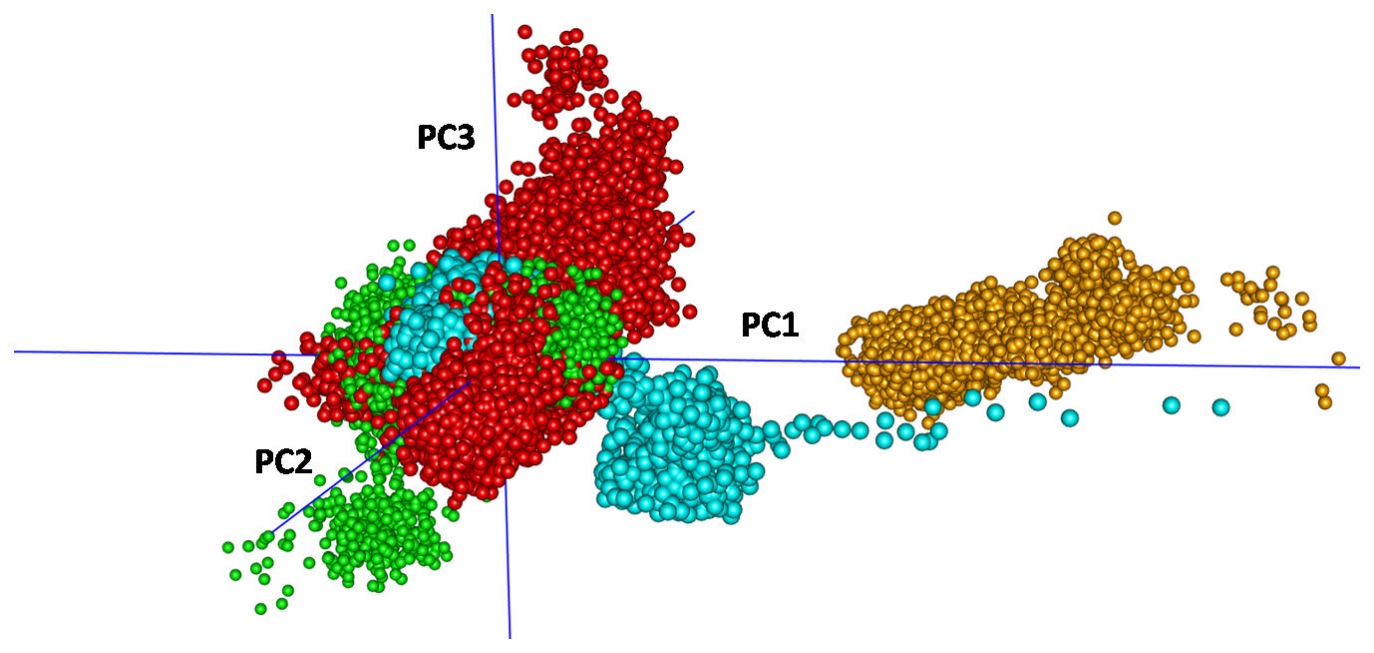
Distribution of the conformations along first three principal components. Different colors were used to show points from different trajectories: Bcl-xl in water (cyan), in membrane (red) and complex in water (green) and in membrane (orange). Also see Movie S7 to view from different sides.

The computed MMGBSW/SA energies from the trajectory of uncomplexed Bcl-xl in water, where the tail folds into its own pocket, shows a ~-45 kcal/mol difference in stability (ΔE^open-closed^) (See Figure S11) between the open and closed states. This is similar to the binding of the tail whose interaction with the protein (as an intermolecular interaction) is ~ -46 kcal/mol (table S1); this intermolecular binding was modelled by simulating the interaction between Bcl-xl (residues 1-217, ∆45-84 as receptor) and a fragment of the tail (i.e. a separate fragment of residues 196-217 as ligand). In contrast to the binding of the tail, the binding energy of the BH3 ^Bak^peptide converges to ~ -90 kcal/mol (for one trajectory, table S2) and ~ -80 kcal/mol (for another independent trajectory, See Figure 9 and Table S3); here the energies of uncomplexed states were averaged over 50-100ns simulations while for the complexes the averages were calculated over each 10ns window (e.g. 0-10ns, 10-20ns, 20-30ns etc.), thus providing the variation of binding energies (See Tables S2-S5 for details). Computations show that the binding affinity of Bcl-xl towards BH3^Bak^ is higher in magnitute (i.e. more stabilizing) compared to that of the tail in water. This suggests why the tail is displaced by the BH3^Bak^ peptide in water [21]. In contrast, in the membrane, a continuous variation and on an average overall destabilization of the binding energy, at -68 kcal/mol (for trajectory 1, table S4) and -54 kcal/mol (for trajectory 2, table S5) is seen (see Figure 10). Here the variation of energy as a function of time correlates with the structures which are undergoing continuous change, i.e. flattening of the binding surface and loosening of receptor-ligand binding (Movie S5). Together this data suggests that after entering the membrane the Bcl-xl+BH3 complex loses its stability. The magnitude of the binding energies obtained from the MMGBSW/SA analysis aparently seems to be overestimated, mainly because our simulations only sample the conformational space around the complexed states. Therefore, the trend of the stabilities of the systems is meaningful and not the absoute quantities. In addition, the solute entropies, particularly those arising from the released waters during the transition from water to the membrane phase will undoubtedly play a role to fine tune the binding free energies; these will be addressed with a more rigorous simulations using explicit water/membrane in a separate study. Garcia-Saez et al. [20] have reported that the BH3-only peptide is capable of dissociating the Bid-Bcl-xl complex in water, but cannot do so in the membrane. Our results are in full agreement with this stronger affinity of BH3 in solution. Garica-Saez et al. [20] also reported that Bcl-xl in membrane exposes hydrophobic residues through conformational changes which helps their stability in the membrane, a feature also seen in our simulations.

### Principal Components

Principal components (PC) of internal motions of the atoms that form the cleft of Bcl-xl (Cα-atoms of 1-196 residues) were calculated for two different sets. The first set combined four different trajectories for the uncomplexed and complexed systems in water and in the membrane, i.e. covering all the conformations accessed by the system; the second set was composed of the subset of conformations from each trajectory separately (inset of Figure S12). All the trajectories were oriented with respect to the uncomplexed Bcl-xl that was averaged over the last 10ns of the simulations in water, with the tail region excluded (owing to the large flexibilities). In the first set, more than 80% of the variance was covered by the first three PCs (see Figure S12), which is in agreement with previous reports on other systems [49,50]. Distributions of the conformations projected along the first three PCs in Figure 10 shows separate clusters originating from the complexed and uncomplexed systems in water and in membrane; the complex in the membrane is well separated from the other systems (Movie S7). From the second set the motions along the PCs 1 and 2 (Movie S8, Movie S9, Movie S10, Movie S11) show that the uncomplexed system in water oscillates (particularly h2, h3) in a manner that tends to cover the binding pocket, with h4 (resi 139-156) largely immobile (Movie S8). In contrast, in the membrane, as shown by the first two PCs, there is considerable movement of h4 which appears to favor the exposure of the core and has correlated twisting modes of h2 and h3 (Movie S9).

#### Simulations in explicit water and membrane

**Figure 11:**
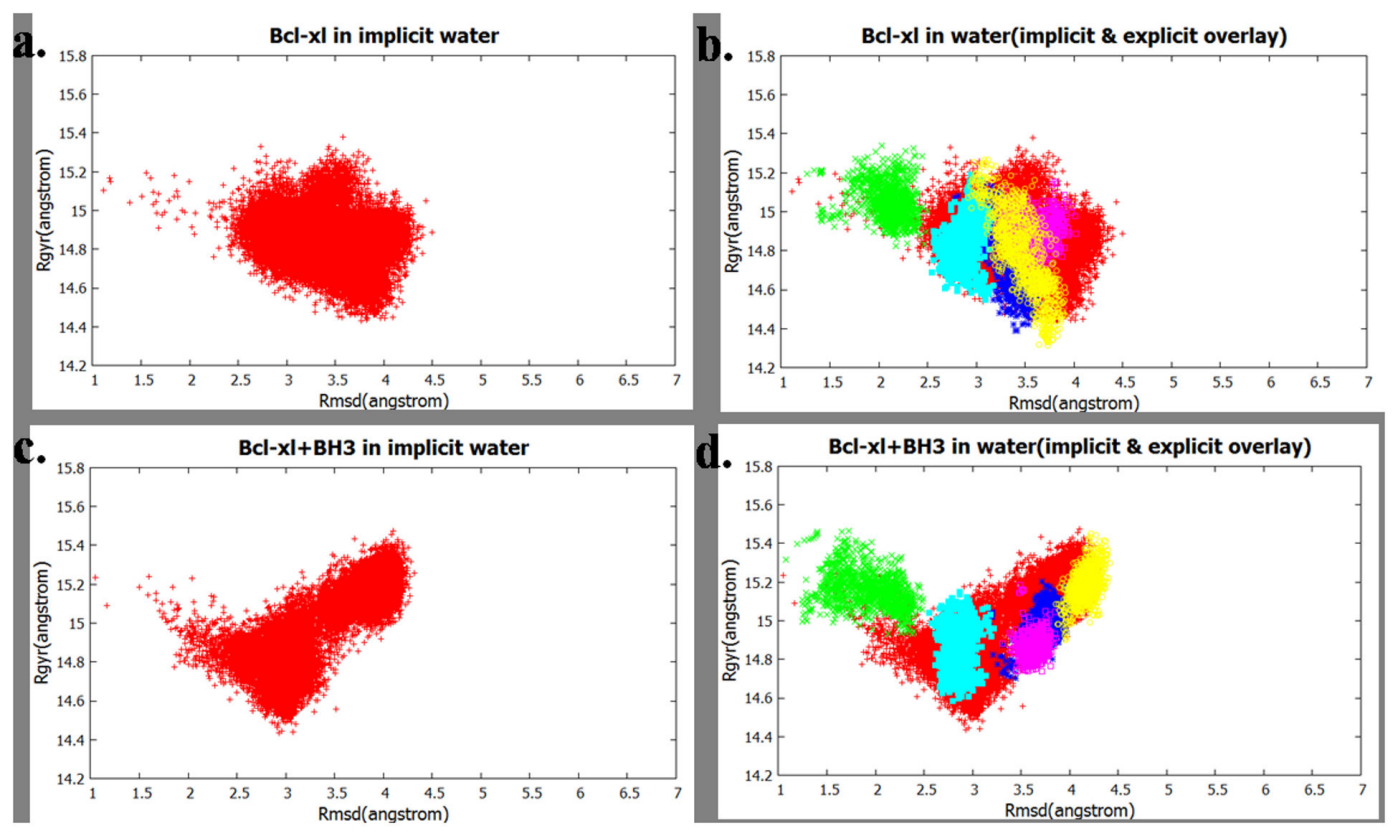
Conformational space sampled in water, shown as a function of RMSD (with respect to the NMR structure) of residues 1-196 vs R_g_. (a) Bcl-xl uncomplexed in implicit water and (b) the uncomplexed Bcl-xl sampled in explicit water overlaid on the points sampled in implicit water ; (c) The conformations of the Bcl-xl in complexed with BH3^Bak^ in implicit water and (d) the same obtained in explicit water overlaid on the points sampled in implicit water. Coloring scheme: red color for the points sampled in implicit medium and five other colors (blue, green, cyan, magenta, yellow) represents sampling in explicit water and different colors correspond to different independent trajectories.

**Figure 12:**
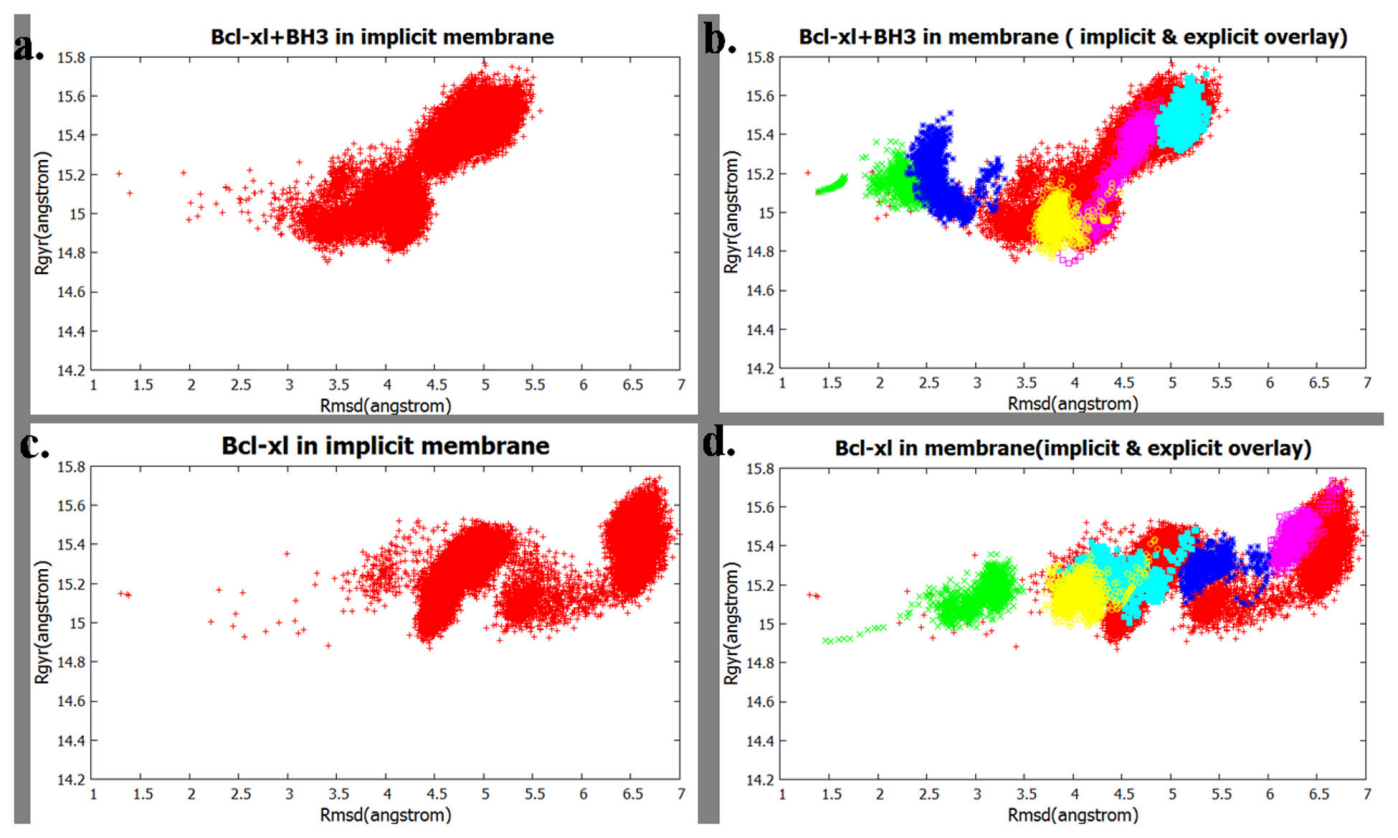
Conformational space sampled in membrane, shown as a function of RMSD (with respect to the NMR structure) of residues 1-196 vs R_g_. (a) Bcl-xl uncomplexed in implicit membrane and (b) the uncomplexed Bcl-xl sampled in explicit membrane overlaid on the points sampled in implicit membrane ; (c) The conformations of the Bcl-xl in complexed with BH3^Bak^ in implicit membrane and (d) the same obtained in explicit water overlaid on the points sampled in implicit membrane. Coloring scheme: same as Figure 11.

Simulations in explicit water and in explicit membrane were carried out starting from randomly selected conformations of proteins generated in corresponding implicit model simulations. The sampled conformational space was presented in a 2D plot using the backbone root mean square deviation (RMSD) of the residues 1-196 (cleft portion) with respect to the NMR structure (1BXL) and radius of gyration(R_g_) of the structures as the two axes. Figure 11a shows the conformational space of uncomplexed Bcl-xl sampled in implcit water and Figure 11b shows the points sampled in the explicit water overlaid on the plot for the impilcit water. Thus it shows that in explicit water environment the sampled space is similar to that observed in the implcit water. The Figure 11c and 11d compare the Bcl-xl+BH3 complex in implicit and explicit water and give the same conclusion. Similarly Figure 12a-d show the sampling for the systems in implcit and explicit membrane, implying that the conformational space accessed in the explcit membrane does not have any significantly different new regions that was not seen from the implcit membrane; rather sampling in implicit environment covers wider area. Overall the structures obtained from implicit simulation are also stable in explicit solvent. The inability of explicit simulation to cover entire conformations of implicit is due to the fact that the presence of explcit collisions makes the sampling speed slower indicated by the densely packed sampled points for each trajectory (shown with different colours in Figure 12). The time required for similar conformational change to take place in explicit environment is much higher compared to the same in implicit solvents.

From the explicit membrane simulation interaction of protein residues with the lipid headgroups and water molecules in the transmembrane region was observed in atomistic detail. In the uncomplexed system the charged/polar residues of the tail (e.g. R206, E208, R209 etc.) and also residues from the cleft (e.g. R100, Q111 etc.) were found to form polar interactions with negatively charged lipid head groups (particularly with the oxygen atoms of the phosphate groups) (see Figure S13). The c-terminal end of the protein was stabilized at the membrane-water reference (Movie S12). On the other side of the membrane N128, R132, D133 were found to be involved in interaction with lipid headgroups (Figure S13). A small stretch of the protein (residue 179-185) was getting stabilized at the surface of the lipid, where polar residues (E179, Q183, E184, N185) remained solvated in water. In BH3 bound Bcl-xl the residues interacting with headgroups are more or less similar (Figure S14 and Movie S13). Residues Q73 and Q77 of Bak were projecting towards the headgroups, which might allow the initiation of weakening of Bcl-xl-BH3 binding (a process that was witnessed in implcit environment).

## Discussion

The Bcl2 family of proteins are found to exist in two regions of conformational space and this appears to be directly relevant to their biology. One region is observed in the solution phase, and has been observed in NMR and x-ray crystallographic studies. Molecular dynamics simulations in water yield conformations that are very similar to the experimentally observed states. However, the region of the conformational space accessed by these proteins when they are in the membrane remains relatively uncharacterized at the atomic level. A few studies in micelles that mimic membranes [51,52] suggest that Bcl-xl is stabilized in micelle by hydrophobic interaction of helices h1, h6, h5 and the c-terminal domain. But it is clear from biochemical studies that the conformations in the membrane are very different from those in water and in membrane it can attain a wide range of conformations. The existence of multiple types of conformations of Bcl-xl in membrane has also been reported experimentally by Billen et al. as ‘functional’ and ‘non-functional’ states [48]. Our current study shows that indeed the conformational ensemble in the membrane differs significantly from that in solution with the microstates in the membrane characterized by exposed hydrophobic cores and complexation capability that is very different from that in the aqueous phase.

In aqueous solution, uncomplexed Bcl-xl undergoes conformational changes that cover the exposed hydrophobic binding pocket (as witnessed in multiple independent simulations), and this is understood to be the most stable conformation in solution in the absence of BH3-only ligands. The tail can bind to this pocket either intramolecularly (as seen in the current study) or intermolecularly, mimicking a dimeric interaction (as shown in recent experiments) [45]. The computed energy difference between the open and closed states is ~ -45 kcal/mol. This stability is attained gradually over 100ns during the simulations (Figure S11). Upon the binding of the tail, the binding pocket also undergoes changes, resulting in a tight complex. This is also seen from principal component analysis where helices h2 and h3 oscillate in a manner that appears favourable for the closure of the binding pocket (see Movies). Simulations triggered from the BH3-peptide complexed Bcl-xl, i.e. starting from the NMR structures, also show a gradual change of the binding energy of BH3 as a function of time during the simulations (see Figure 9), resulting in high affinity (~-90 kcal/mol) compared to that of the tail, in agreement with experiments. The movements of the Bcl-xl cleft along the principal components are also similar for Bcl-xl complexes with the tail or with BH3, suggesting that the internal motions along the PCs are similar in water and this helps to maintain tight packing at the binding pocket.

In the membrane, the uncomplexed Bcl-xl expands and exposes the hydrophobic binding pocket (See Movie S4 shown with different colours for the binding pocket residues). The average structures and fluctuations along the PCs are very different from that in water. The binding energies (Figure 9) show that the ligand does not bind stably to the protein in the membrane compared to that in solution. Together, the data suggests that BH3 can displace the tail from the binding pocket of Bcl-xl and can form a stable complex in aqueous phase, but in the membrane, this complex loses its stability through large conformational changes, particularly around the binding pocket. This is no longer suitable for gripping a peptide, and the larger binding area now appears more suitable for complexing with a protein that also has a large exposed surface. To check whether the exposed binding pocket of Bcl-xl is really able to bind full length Bak, a preliminary study was done (data not shown). The coordinates of full length Bak were obtained from its crystal structure (RCSB entry 2IMT [13]) and were subject to simulation for 10ns in an implicit membrane environment to examine the conformational changes. Then final conformation from this simulation was docked to the final conformation of Bcl-xl (uncomplexed, see Table 1) from its 100ns simulation in implicit membrane. The program Patchdock [26] was used to dock the two conformations and the complex with minimum binding free energy (i.e. most stable association) was selected. These two proteins are observed to interact through their large exposed binding surfaces in the membrane. During the preliminary simulation (20ns) of this complex (BXL ^mem^Bak) in the membrane, the complex remained stable and a representative snapshot has been shown in supporting information (Figure S15). We repeated these steps of modeling and simulation in implicit membrane with the starting structure taken from the NMR structure 1BXL of Bcl-xl. The complex (BXL ^nmr^Bak) shows reduced stability (data not shown) compared to BXL ^mem^Bak and and also much weaker van der Waals interactions (-155.75 kcal/mol for BXL ^nmr^Bak and -214.16 kcal/mol BXL ^mem^Bak) between the two partners. This indicated a much stronger association of the partners in BXL ^mem^Bak confirming that the Bcl-xl with largely exposed hydrophobic surface has higher affinity for Bak in the membrane. A similar trend is observed in the total energy of the system. The surface electrostatic potential calculated in vacuum shows that hydrophobic-hydrophobic contacts upon complexation are larger for BXL ^mem^Bak compared to BXL ^nmr^Bak (see Figure S16). It is believed that once the tail is freed by the complexation of BH3, it anchors in the membrane, which helps the insertion of Bcl-xl in the membrane. Preliminary results (data not shown) of our simulations have shown such anchoring, however these processes are expected to occur at much longer timescales (plausibly in the order of hundreds of microseconds to milliseconds) and hence are beyond the scope of the current study.

There are no biologically relevant experimentally determined structural coordinates for this (Bcl2 family members) system available. The X-ray solution NMR data have been determined under aqueous conditions and hence do not reflect the biologically relevant structural ensemble, as the biology takes place in the membrane. Hence it is appropriate to use MD simulations of the system in an environment that mimicks the membrane to develop hypotheses regarding the molecular mechanisms underlying the function. Of course, given that the time covered by these simulations is short, although the conformations from multiple independent simulations agree with each, nevertheless it is possible that at a much longer timsecale e.g. in milli- to micro-second, the systems in the membrane can elaborate its conformational search depending on its functional need. Indeed the conformation of Bcl-xl in membrane may not be unique, it can possibly access a loosely packed conformational space when it is uncomplexed, i.e. can access a much wider conformational space. This is particularly needed because the system has to bind a multitude of proapoptotic partners with different shapes, and hence the need to change shapes to accommodate this easily will benefit from a loosely packed apo state as proposed from NMR studies by Losonczi et al. [51] We do see such loosening of packing in our simulations as can be seen from changes (from water to membrane) in the principal coponent movies that show that the dominating movements appear to fascilitate the closure of the binding pocket in water, whereas the dominant modes in the membrane appear to assist in the opening of the binding pocket. The projection of trajectories along first three PCs shows (Figure 10) that the conformations of the uncomplexed Bcl-xl in mebrane cover a much wider region of conformational space compared to the same system in water. The Bcl-xl-BH3^Bak^ complex also forms a distinct cluster in the PC-space which is much separated from the other systems. Thus the primary membrane simulations have revealed that the dynamics of the protein in the membrane are necessarily altered to enable the system to choose its biological partner in the membrane.

## Conclusions

Computer simulations have been used to study, in atomistic detail, the difference of the dynamics of Bcl-xl in membrane and in water and complement the emerging picture from other experimental and computational studies [53-56]. The ability to use an implicit model of a membrane, through the use of the GBSW-membrane model [34,57] has proven to be very useful in enhancing the sampling speed with cost-effective use of CPUs. Although implcit models may often overestimate the polar interactions, GBSW is reported to be very accurate [58] compared to the corresponding Poisson-Boltzmann (PB) calculations but executed at a much lesser computing time. Recently, Pang et al. [54] demonstrated how octamer formation of Bak could be induced by a process of stepwise oligimerization. This was demonstrated in the water phase which mimics the cytoplasmic phase, whereas the biology of the pore formation and its structural stability is relevant only in the mitochondrial membrane. Pang et al. [54] observed that at least in solvent, little conformational change was necessary for Bak to oligomerize. In contrast, our studies show that in the membrane, a large departure takes place from the structure of Bcl-xl that binds a small BH3 peptide in water. An exposure and flattening of the binding pocket occurs, presumably making it receptive to binding another protein. Our simulations show that in water the tail is the self inhibitor which may also help in dimerization, as observed from experimental reports [45]. The BH3^Bak^ domain peptide (has sequence similarity with other BH3-only peptides) competes for binding to Bcl-xl and has the ability to replace the tail from the binding groove. Reconciling this with experimental data [19,59] it could be proposed that once the tail is free, it is available for anchoring into the membrane. As the next step of the mechanism, simulations in the membrane show a reduced binding affinity of the BH3^Bak^ compared to that in water, which allows the release of the BH3-peptide in order to bind to another protein to form a dimer. In the time series of binding energies (Figure 9), there is an overlapping region for the systems in water and in the membrane (i.e. BH3^Bak^ is equally stable in membrane and water in those conformations). This may reflect a region of the conformational space where transfer from membrane to water (or the reverse) occurs, thus maintaining an equilibrium. Once the complex is transferred to the membrane, the peptide loses its binding affinity as Bcl-xl undergoes a conformational change, resulting in the transfer of the peptide back to the aqueous phase, as proposed in the ‘hit-and-run’ mechanism[21]. Simulations have also revealed the inherent flexibility of the binding pocket of Bcl-xl. The promising Bcl2-class inhibitors (e.g. ABT737, ABT263, TW-37 etc.) [9,11] may be refined considering the plasticity of the binding pocket to be target specific in future design of novel targeted therapeutics [60-62].

## Supporting Information

Text S1 **Details of the methodologies of the simulations using explicit water and membrane.**
(DOC)

Figure S1 **Covering of the binding pocket by the C-terminal tail.** The tail shown in red is covering the hydrophobic residues that are forming the binding pocket in the nMR structure (1BXL) shown by blue patches.
(TIF)

Figure S2 **Different orientations of tail in two independent trajectory of uncomplexed Bcl-xl in water.** The cartoon in cyan is representing helix h3(resid 120-130) and tail is represented in red.
(TIF)

Figure S3 **Orientation of tail of complexed bcl-xl in water in trajectory 2.** The tail (in red) is orienting itself at the lower part of the cleft.
(TIF)

Figure S4 **Salt bridge interactions (D83^Bak^:R139^Bcl-xl^ and R76^Bak^:E129^Bcl-xl^) from simulation which are also reported in the NMR structure.** Bcl-xl cleft (residue 1 to 196), c-terminal tail (residue 197 to 217) and BH3^Bak^ are shown in green, red and blue cartoon respectively. Hydrogen bond between the residues, shown in stick are shown in blue dashes.
(TIF)

Figure S5 **Distance between two pair of residues glu221:arg103 and leu210:phe146 over the simulation time period of bcl-xl in water and membrane over 95 ns of simulation.**
(TIF)

Figure S6 **(a) Root mean square deviation (Rmsd) and (b) Radius of Gyration (Rgyr) of backbone atoms of the cleft (residue 1-196 of Bcl-xl) for different systems.**
(TIF)

Figure S7 **Solvent accessible surface area of the structures obtained after 100ns simulations of uncomplexed Bcl-xl in water and in membrane.** The accessibility of the polar and non-polar residues has been shown separately.
(TIF)

Figure S8 **Different orientations of tail in two independent trajectory of uncomplexed Bcl-xl in membrane.** The cartoon in cyan is representing helix h2(resid 85-101) and tail is represented in red.
(TIF)

Figure S9 **Structure of Bcl-xl+BH3^Bak^ complex in membrane after 100 ns of simulation: residue involved in a) salt bridge interaction and b) hydrophobic interactions are shown in stick.** Hydrogen bond among charged side chains are shown in blue dash.
(TIF)

Figure S10 **Orientation of tail along membrane axis driven by salt bridge interaction among polar side chains of R100, R204 and D584.** After 10 ns the c-terminal histidines (H212, H213) are getting oriented in the region with higher polarity (described as switching region in implicit membrane) being attracted by side chain of R103.
(TIF)

Figure S11 **Plot of total energy (EMM) of the Bcl-xl along the trajectory, showing the difference of tail-closed and tail-open states.**
(TIF)

Figure S12 **Plot of cumulative sum percentage of variance as a function of PC rank for the modes calculated from the sum of four trajectories (Bcl-xl and Bcl-xl + BH3^Bak^ in water and membrane).** Inset shows the PC calculations from separate trajectories.
(TIF)

Figure S13 **Proposed structure of full length Bcl-xl and Bak complex simulated in implicit membrane.**
(TIF)

Figure S14 **Vacuum electrostatic of Bcl-xl-Bak complex; a) larger hydrophodic compatibility between the surfaces of Bcl-xl^mem^ and Bak, b) lesser hydrophobic compatibility between Bcl-xl^nmr^ and Bak.** Colour codes: negetive (Red), positive (Blue), neutral (White).
(TIF)

Figure S15 **A model of the complex of full length Bcl-xl and Bak.** The snapshot was taken at the end of 20ns simulation in implicit membrane.
(TIF)

Figure S16 **Surface electrostatics of Bcl-xl-Bak complexes: (a) Bcl-xl^mem^ and Bak, (b) Bcl-xl^nmr^ and Bak.** Colour codes: negetive (Red), positive (Blue), neutral (White). Aroows indicate the areas on the surface which are in contact with each other when the compelx is formed.
(TIF)

Movie S1 **Dynamics of Bcl-xl in implicit water.**
(ZIP)

Movie S2 **Dynamics of the Bcl-xl and BH3 complex in implicit water.**
(ZIP)

Movie S3 **Dynamics showing the alignment of Bcl-xl along membrane (implicit).** axis.
(ZIP)

Movie S4 **Dynamics of Bcl-xl inimplicit membrane.**
(ZIP)

Movie S5 **Dynamics of the complex in implicit membrane.**
(ZIP)

Movie S6 **Concerted hydrophobic collision to kick out BH3 from Bcl-xl.**
(ZIP)

Movie S7 **3D plot of first three principal components for receptor and complexes in different environment.**
(ZIP)

Movie S8 **Fluctuation along first three principal components of Bcl-xl in water.**
(ZIP)

Movie S9 **Fluctuation along first three principal components of bcl-xl in membrane.**
(ZIP)

Movie S10 **Fluctuation along first three principal components of bcl-xl+BH3 in water.**
(ZIP)

Movie S11 **Fluctuation along first three principal components of bcl-xl+BH3 in membrane.**
(ZIP)

Movie S12 **Dynamics of Bcl-xl in explicit membrane.**
(ZIP)

Movie S13 **Dynamics of the complex in explicit membrane.**
(ZIP)

Table S1 **Calculated binding energy (in Kcal/mol) of tail (resid 197-217 of Bcl-xl) with bcl-xl in implicit water.** Energies of each bcl-xl+bak, bcl-xl and bak are averaged from 5-30 ns of simulation.
(DOC)

Table S2 **Calculated binding energy (in Kcal/mol) of Bh3^bak^ with Bcl-xl at different time window.** E _Bcl-xl + Bak_ is the energy of complex in water averaged over the particular window of time in the independent trajectory no. 1. E _Bcl-xl_ and E_Bak_ are energies of respective molecules in water averaged over last 50-100 ns simulation.
(DOC)

Table S3 **Calculated binding energy (in Kcal/mol) of Bh3^bak^ with Bcl-xl at different time window.** E _Bcl-xl + Bak_ is the energy of complex in water averaged over the particular window of time the independent trajectory no. 2. E _Bcl-xl_ and E_Bak_ are energies of respective molecules in water averaged over last 50-100 ns simulation.
(DOC)

Table S4 **Calculated binding energy (in Kcal/mol) of BH3^Bak^ with Bcl-xl at different time window.** E _Bcl-xl + Bak_ is the energy of complex in membrane averaged over the particular window of time in the independent trajectory no. 1. E _Bcl-xl_ and E_Bak_ are energies of respective molecules in water averaged over last 50-100 ns simulation.
(DOC)

Table S5 **Calculated binding energy (in Kcal/mol) of BH3^Bak^ with Bcl-xl at different time window.** E _Bcl-xl + Bak_ is the energy of complex in membrane averaged over the particular window of time in the independent trajectory no. 2. E _Bcl-xl_ and E_Bak_ are energies of respective molecules in water averaged over last 50-100 ns simulation.
(DOC)

Table S6 **List of trajectories simulated using explicit water and explicit membrane environment.**
(DOC)
