# Supplementary material for: Dynamics of Bcl-xL in Water and Membrane: Molecular Simulations
Source: PLoS One. 2013 Oct 8;8(10):e76837. doi: 10.1371/journal.pone.0076837 (PMC3792877; doi:10.1371/journal.pone.0076837)
Supplement: Text S1 — Details of the methodologies of the simulations using explicit water and membrane. (DOC) [file pone.0076837.s001.doc]

**Details of the methods of simulations in explicit water and membrane:**

**Explicit water:**

Each of the representative coordinates taken from implicit water simulation was solvated in a cubic box of TIP3P water. The size of the box was determined by maintaining a minimum distance 9 Å from the edge of the protein. To maintain experimental condition adequate number of potassium and chloride ions were added to reach 0.15 (M) salt concentration. Each system was minimised 5000 step using both ABNR (Adopted Basis Newton-Raphson) and SD (Steepest Descent) algorithm with constraint on backbone atoms using CHARMM22 forcefield and parameters with CMAP correction, i.e. the same as implicit water/membrane simulations. Then these systems were subjected to heating and then 1ns equilibration using NVT ensemble at 300K. Then 10 ns Langevine dynamics (Langevin damping coefficient of 1/ps) under NPT condition was performed for each trajectory in NAMD simulation engine with temperature 300K and pressure 1 atm maintained using Nose-Hoover thermostat/barostat. For the calculations of short ranged non-bonded interaction a cutoff value of 12 Å was used, whereas the long range electrostatics was treated using Particle Mesh Ewald technique. SHAKE was applied to freeze the vibrations of bonds involving hydrogen to allow 2fs integration timestep

**Explicit membrane:**

All representative coordinates chosen from implicit membrane simulation were embedded in a prequilibrated DOPC (1,2-Dioleoyl-*sn*-glycero-3-phosphocholine) bilayer using charmm-gui membrane builder web-server . The number of lipid molecule in upper and lower leaflet varied from 90 to 110 depending on the system size. 25 Å TIP3P water layer was maintained at each side over the lipid headgroups. Potassium and chloride ions were added to maintain 0.15 (M) salt concentrations. The force field was used as similar to explicit water simulation, except the lipid parameters came from CHARMM27. Protein was minimized stepwise within the bilayer using a harmonic restraint on lipid and protein backbone using the same algorithm as used for water. Rest of the scheme was same as explicit water simulations, including the trajectory length.

**References:**

1. Phillips, J.C., et al., *Scalable molecular dynamics with NAMD.* J Comput Chem, 2005. **26**(16): p. 1781-802.

2. Jo, S., et al., *CHARMM-GUI Membrane Builder for mixed bilayers and its application to yeast membranes.* Biophys J, 2009. **97**(1): p. 50-8.
