# Supplementary figures and images for: Dynamics of Bcl-xL in Water and Membrane: Molecular Simulations
Source: PLoS One. 2013 Oct 8;8(10):e76837. doi: 10.1371/journal.pone.0076837 (PMC3792877; doi:10.1371/journal.pone.0076837)

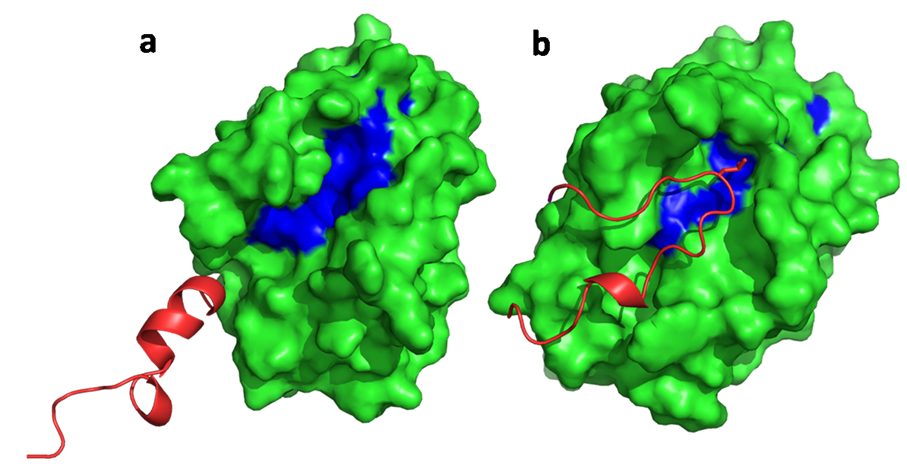

Supplement: Figure S1 — Covering of the binding pocket by the C-terminal tail. The tail shown in red is covering the hydrophobic residues that are forming the binding pocket in the nMR structure (1BXL) shown by blue patches. (TIF) [file pone.0076837.s002.tif]

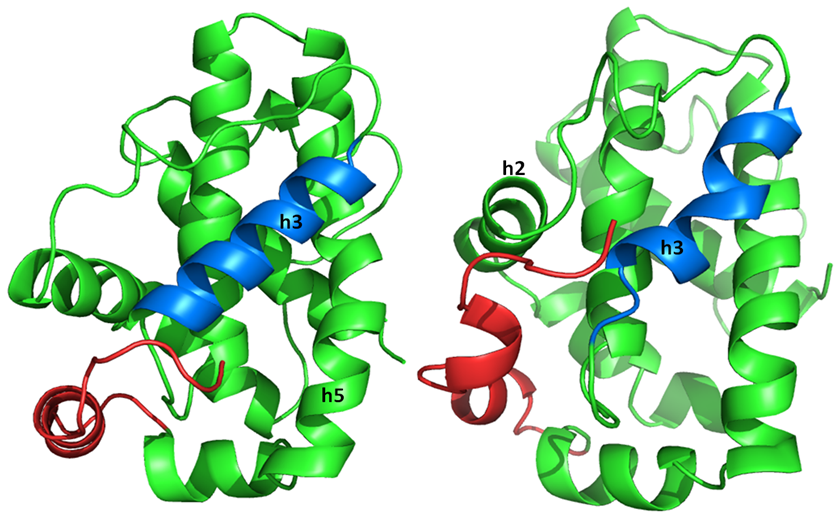

Supplement: Figure S2 — Different orientations of tail in two independent trajectory of uncomplexed Bcl-xl in water. The cartoon in cyan is representing helix h3(resid 120-130) and tail is represented in red. (TIF) [file pone.0076837.s003.tif]

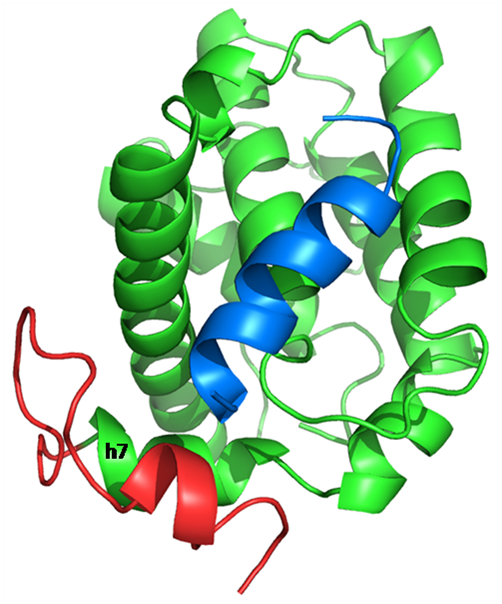

Supplement: Figure S3 — Orientation of tail of complexed bcl-xl in water in trajectory 2. The tail (in red) is orienting itself at the lower part of the cleft. (TIF) [file pone.0076837.s004.tif]

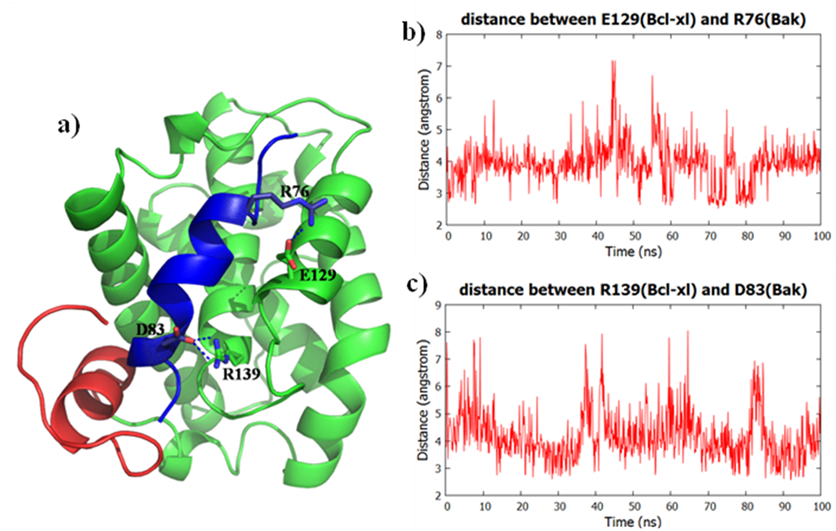

Supplement: Figure S4 — Salt bridge interactions (D83Bak:R139Bcl-xl and R76Bak:E129Bcl-xl) from simulation which are also reported in the NMR structure. Bcl-xl cleft (residue 1 to 196), c-terminal tail (residue 197 to 217) and BH3Bak are shown in green, red and blue cartoon respectively. Hydrogen bond between the residues, shown in stick are shown in blue dashes. (TIF) [file pone.0076837.s005.tif]

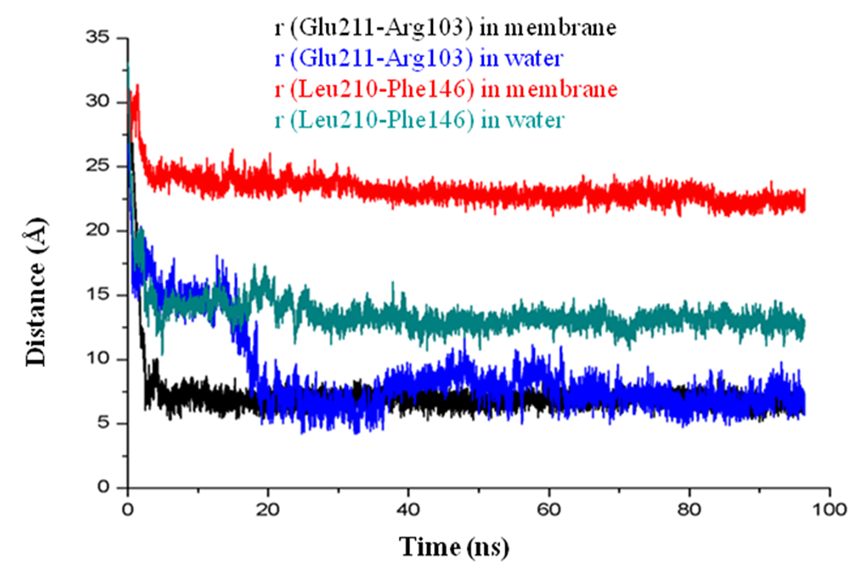

Supplement: Figure S5 — Distance between two pair of residues glu221:arg103 and leu210:phe146 over the simulation time period of bcl-xl in water and membrane over 95 ns of simulation. (TIF) [file pone.0076837.s006.tif]

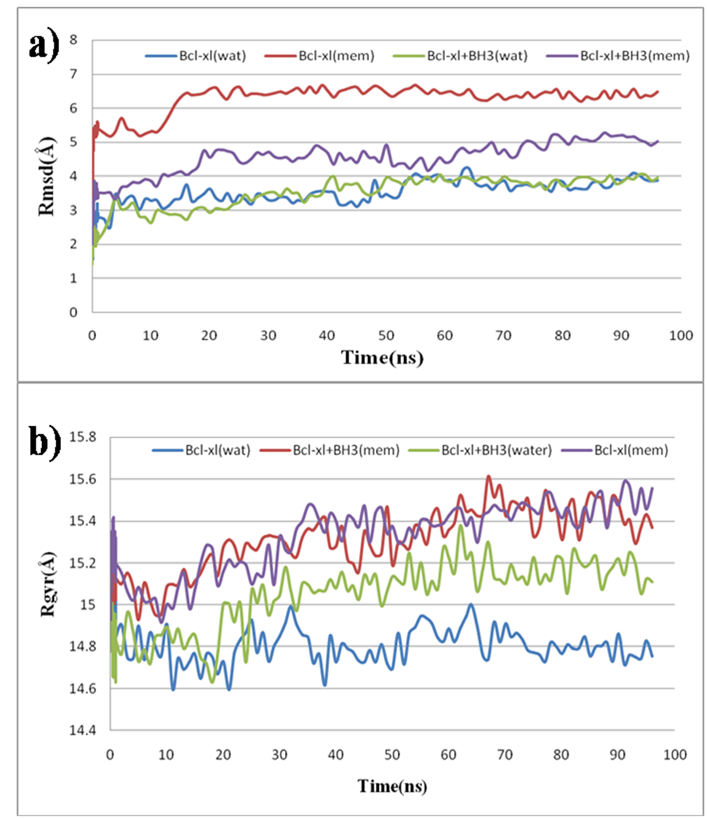

Supplement: Figure S6 — (a) Root mean square deviation (Rmsd) and (b) Radius of Gyration (Rgyr) of backbone atoms of the cleft (residue 1-196 of Bcl-xl) for different systems. (TIF) [file pone.0076837.s007.tif]

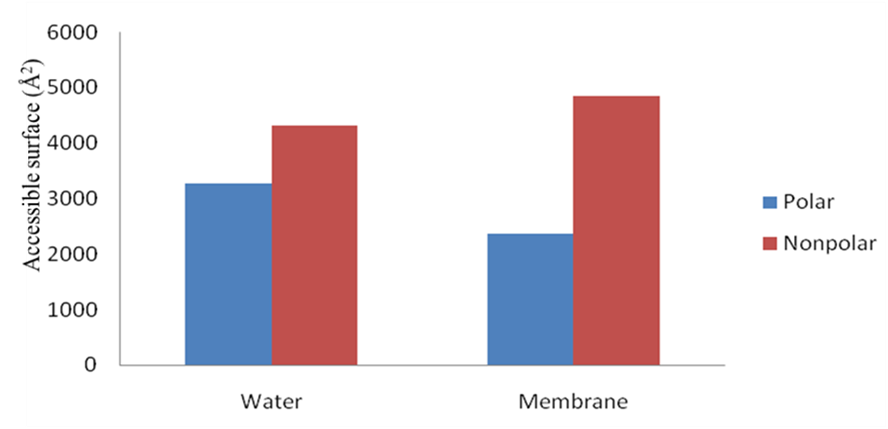

Supplement: Figure S7 — Solvent accessible surface area of the structures obtained after 100ns simulations of uncomplexed Bcl-xl in water and in membrane. The accessibility of the polar and non-polar residues has been shown separately. (TIF) [file pone.0076837.s008.tif]

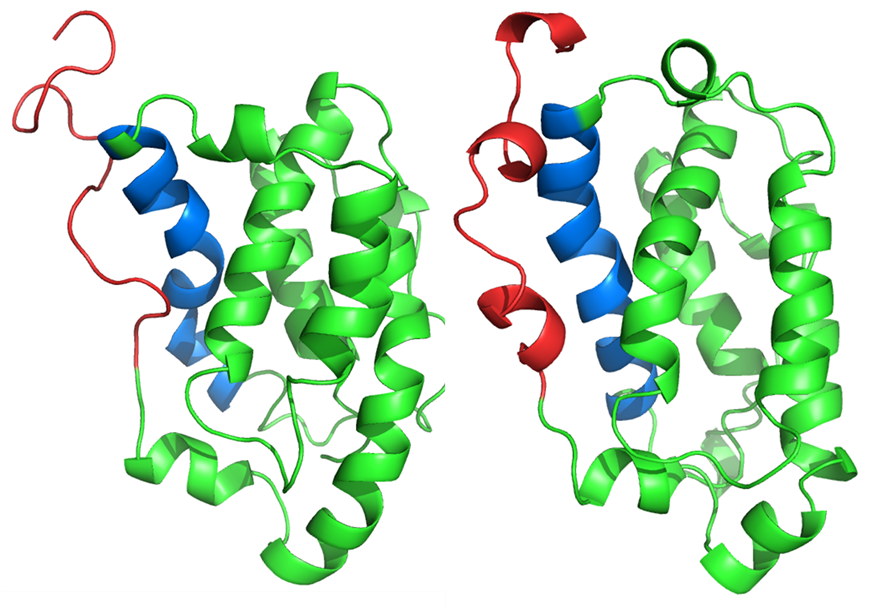

Supplement: Figure S8 — Different orientations of tail in two independent trajectory of uncomplexed Bcl-xl in membrane. The cartoon in cyan is representing helix h2(resid 85-101) and tail is represented in red. (TIF) [file pone.0076837.s009.tif]

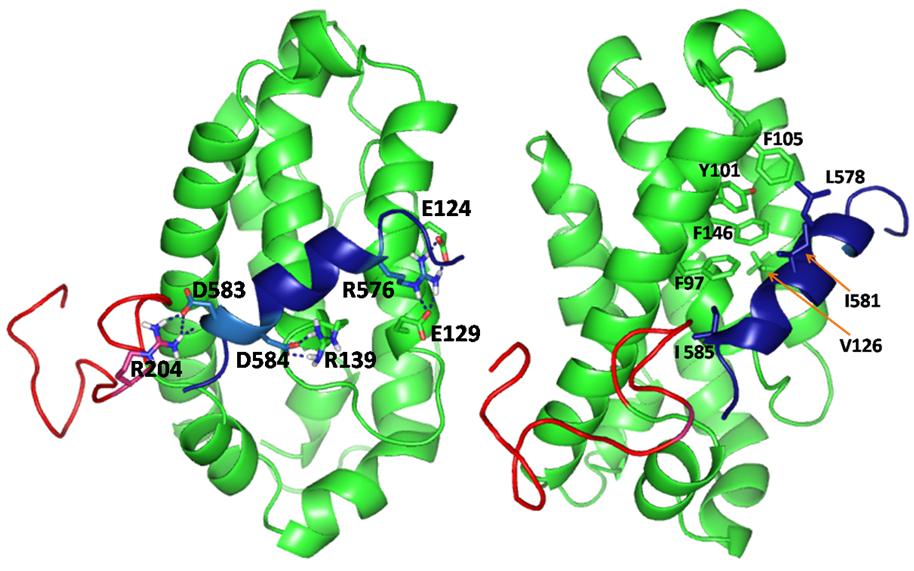

Supplement: Figure S9 — Structure of Bcl-xl+BH3Bak complex in membrane after 100 ns of simulation: residue involved in a) salt bridge interaction and b) hydrophobic interactions are shown in stick. Hydrogen bond among charged side chains are shown in blue dash. (TIF) [file pone.0076837.s010.tif]

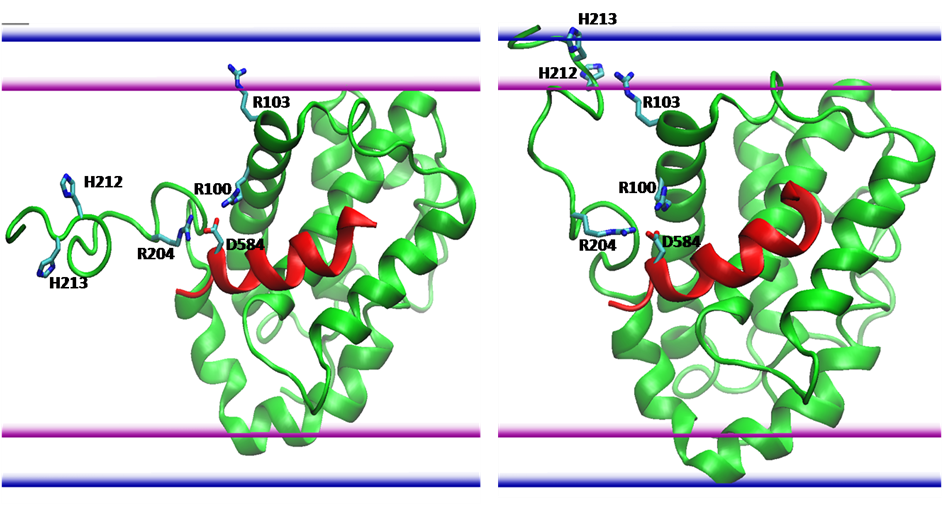

Supplement: Figure S10 — Orientation of tail along membrane axis driven by salt bridge interaction among polar side chains of R100, R204 and D584. After 10 ns the c-terminal histidines (H212, H213) are getting oriented in the region with higher polarity (described as switching region in implicit membrane) being attracted by side chain of R103. (TIF) [file pone.0076837.s011.tif]

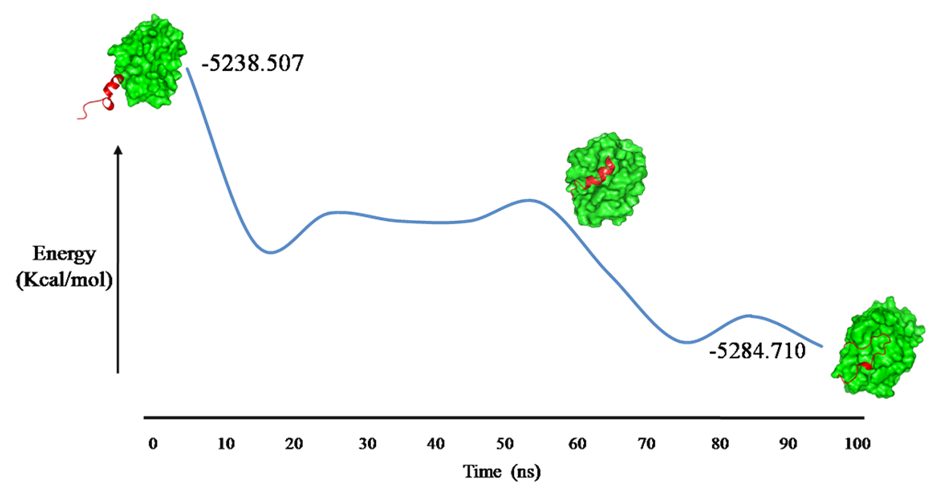

Supplement: Figure S11 — Plot of total energy (EMM) of the Bcl-xl along the trajectory, showing the difference of tail-closed and tail-open states. (TIF) [file pone.0076837.s012.tif]

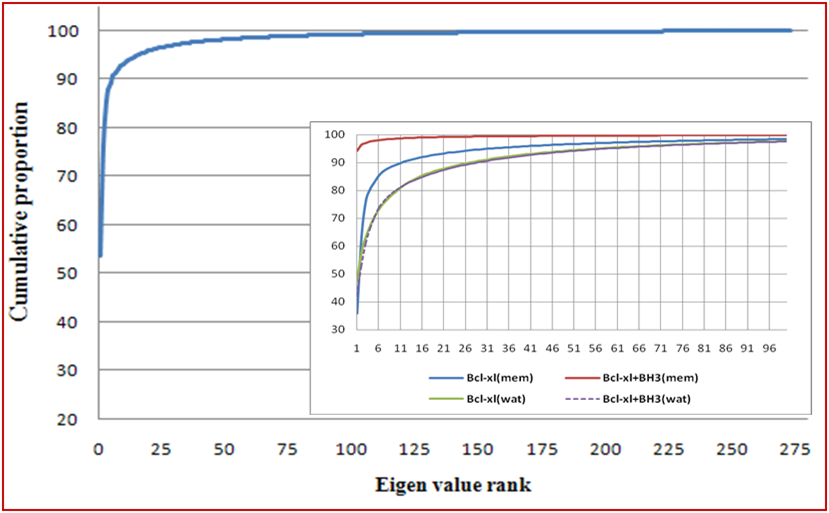

Supplement: Figure S12 — Plot of cumulative sum percentage of variance as a function of PC rank for the modes calculated from the sum of four trajectories (Bcl-xl and Bcl-xl + BH3Bak in water and membrane). Inset shows the PC calculations from separate trajectories. (TIF) [file pone.0076837.s013.tif]

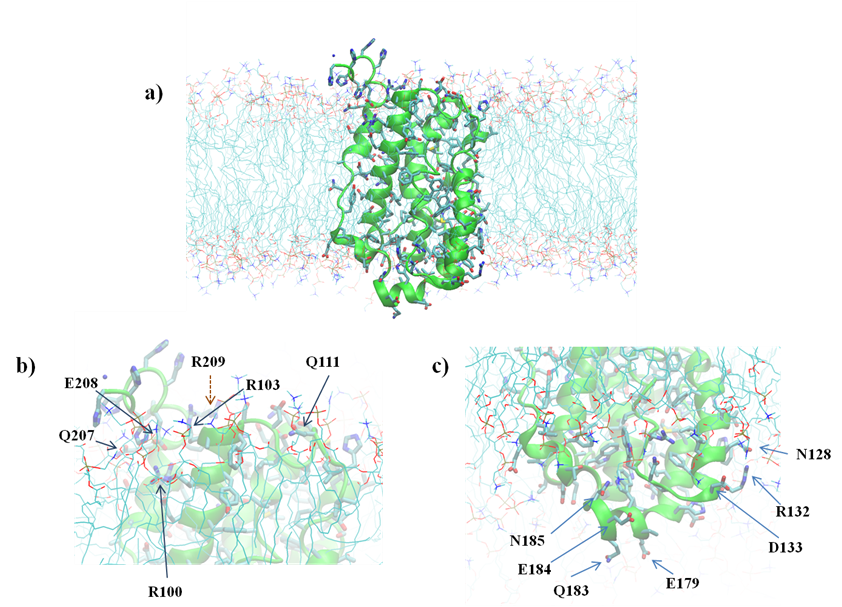

Supplement: Figure S13 — Proposed structure of full length Bcl-xl and Bak complex simulated in implicit membrane. (TIF) [file pone.0076837.s014.tif]

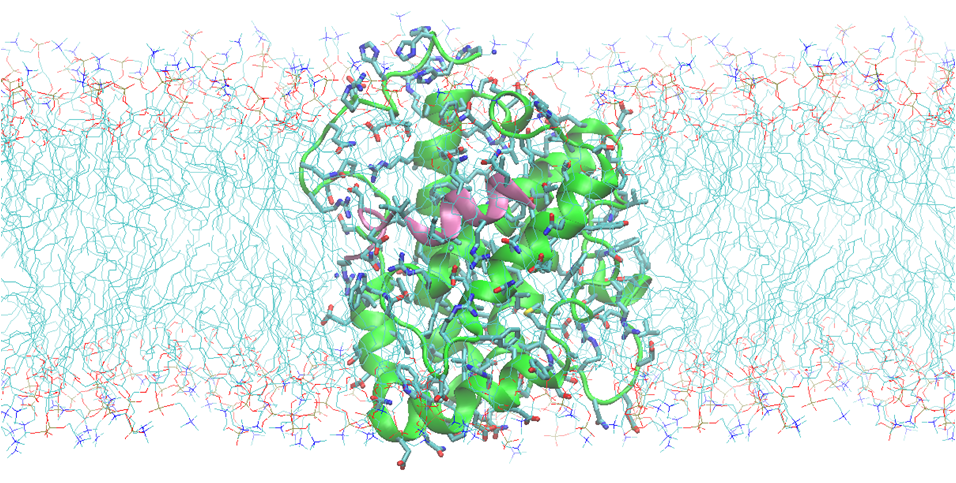

Supplement: Figure S14 — Vacuum electrostatic of Bcl-xl-Bak complex; a) larger hydrophodic compatibility between the surfaces of Bcl-xlmem and Bak, b) lesser hydrophobic compatibility between Bcl-xlnmr and Bak. Colour codes: negetive (Red), positive (Blue), neutral (White). (TIF) [file pone.0076837.s015.tif]

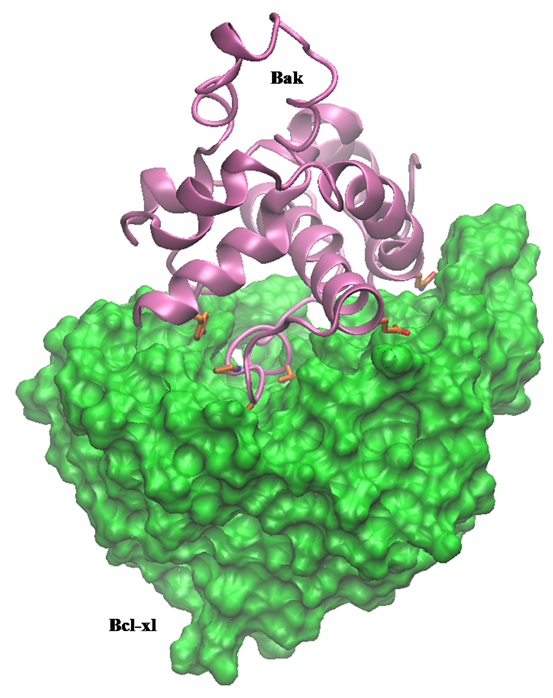

Supplement: Figure S15 — A model of the complex of full length Bcl-xl and Bak. The snapshot was taken at the end of 20ns simulation in implicit membrane. (TIF) [file pone.0076837.s016.tif]

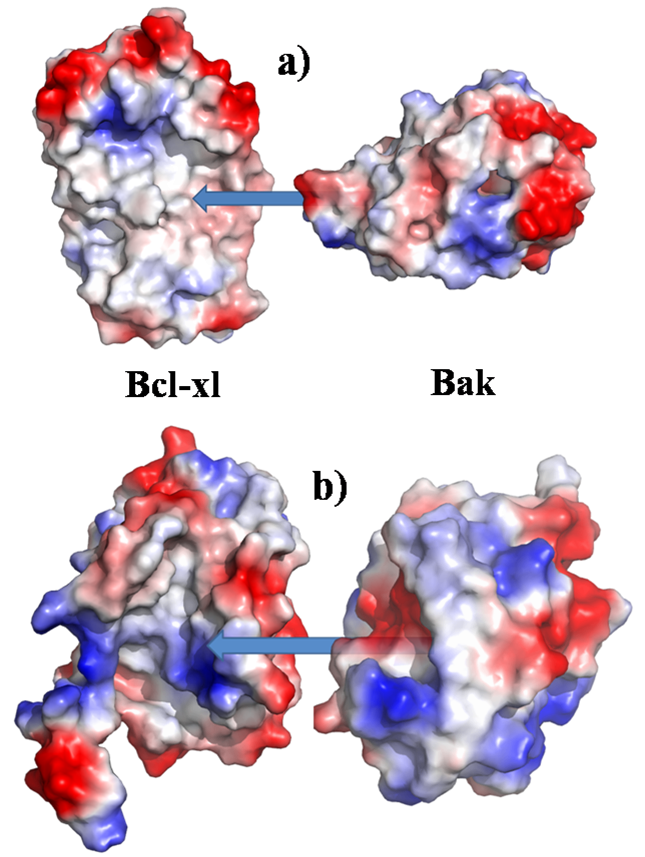

Supplement: Figure S16 — Surface electrostatics of Bcl-xl-Bak complexes: (a) Bcl-xlmem and Bak, (b) Bcl-xlnmr and Bak. Colour codes: negetive (Red), positive (Blue), neutral (White). Aroows indicate the areas on the surface which are in contact with each other when the compelx is formed. (TIF) [file pone.0076837.s017.tif]
