# Supplementary material for: Dynamics of Bcl-xL in Water and Membrane: Molecular Simulations
Source: PLoS One. 2013 Oct 8;8(10):e76837. doi: 10.1371/journal.pone.0076837 (PMC3792877; doi:10.1371/journal.pone.0076837)
Supplement: Table S1 — Calculated binding energy (in Kcal/mol) of tail (resid 197-217 of Bcl-xl) with bcl-xl in implicit water. Energies of each bcl-xl+bak, bcl-xl and bak are averaged from 5-30 ns of simulation. (DOC) [file pone.0076837.s031.doc]

**Binding energy of Bcl-xl with its C-terminal tail**

| **Components of energy** | **E _Bcl-xl + tail_** | **E _Bcl-xl_** | **E_tail_** | **∆E _Binding_** |
| --- | --- | --- | --- | --- |
| E_ELEC_ | -4554.9 | -3619.42 | -871.431 | -64.0457 |
| E_VDW_ | -856.604 | -710.475 | -75.8806 | -70.2484 |
| E_INTER_ | 3253.2 | 2897.157 | 342.252 | 13.79133 |
| E_GAS_ | -2158.33 | -1432.69 | -451.107 | -274.53 |
| E_SOLP_ | -4226.41 | -3907.87 | -548.456 | 229.916 |
| E_NONP_ | 74.8372 | 65.4935 | 11.23135 | -1.88765 |
| E_SOLV_ | -4151.57 | -3842.37 | -537.225 | 228.0283 |
| E_GBELEC_ | -8781.31 | -7527.29 | -1265.94 | 11.92333 |
| E_MM(TOT.)_ | -6309.9 | -5275.07 | -988.332 | -46.5013 |
