# Supplementary material for: Dynamics of Bcl-xL in Water and Membrane: Molecular Simulations
Source: PLoS One. 2013 Oct 8;8(10):e76837. doi: 10.1371/journal.pone.0076837 (PMC3792877; doi:10.1371/journal.pone.0076837)
Supplement: Table S2 — Calculated binding energy (in Kcal/mol) of Bh3bak with Bcl-xl at different time window. E Bcl-xl + Bak is the energy of complex in water averaged over the particular window of time in the independent trajectory no. 1. E Bcl-xl and EBak are energies of respective molecules in water averaged over last 50-100 ns simulation. (DOC) [file pone.0076837.s032.doc]

**Binding energy of BH3^bak^ with Bcl-xl at different time window in trajectory 1 in water**

| **Window of time(ns)** | **E _Bcl-xl + BH3_** ^bak^ | **E _Bcl-xl_** | **E_BH3_** ^bak^ | **∆E_Binding_** |
| --- | --- | --- | --- | --- |
| 0-10 | -6136.47 | -5280.3 | -828.74 | -27.43071 |
| 10-20 | -6165.06 |  |  | -56.02027 |
| 20-30 | -6160.29 |  |  | -51.25268 |
| 30-40 | -6176.51 |  |  | -67.46555 |
| 40-50 | -6178.44 |  |  | -69.40238 |
| 50-60 | -6191.03 |  |  | -81.98976 |
| 60-70 | -6197.46 |  |  | -88.41538 |
| 70-80 | -6194.01 |  |  | -84.96807 |
| 80-90 | -6193.55 |  |  | -84.51402 |
| 90-100 | -6200.17 |  |  | -91.12967 |
