# Supplementary material for: Dynamics of Bcl-xL in Water and Membrane: Molecular Simulations
Source: PLoS One. 2013 Oct 8;8(10):e76837. doi: 10.1371/journal.pone.0076837 (PMC3792877; doi:10.1371/journal.pone.0076837)
Supplement: Table S3 — Calculated binding energy (in Kcal/mol) of Bh3bak with Bcl-xl at different time window. E Bcl-xl + Bak is the energy of complex in water averaged over the particular window of time the independent trajectory no. 2. E Bcl-xl and EBak are energies of respective molecules in water averaged over last 50-100 ns simulation. (DOC) [file pone.0076837.s033.doc]

**Binding energy of BH3^bak^ with Bcl-xl at different time window in trajectory 2 in water**

| **Window of time(ns)** | **E _Bcl-xl + BH3_** ^bak^ | **E _Bcl-xl_** | **E_BH3_** ^bak^ | **∆E_Binding_** |
| --- | --- | --- | --- | --- |
| 0-10 | -6141.25 | -5280.3 | -828.74 | -32.1562 |
| 10-20 | -6152.11 |  |  | -43.074 |
| 20-30 | -6155.76 |  |  | -46.7182 |
| 30-40 | -6174.33 |  |  | -65.293 |
| 40-50 | -6171.47 |  |  | -62.433 |
| 50-60 | -6170.80 |  |  | -61.762 |
| 60-70 | -6181.91 |  |  | -72.866 |
| 70-80 | -6179.28 |  |  | -70.237 |
| 80-90 | -6176.58 |  |  | -67.545 |
| 90-100 | -6190.93 |  |  | -81.892 |
