# Supplementary material for: Dynamics of Bcl-xL in Water and Membrane: Molecular Simulations
Source: PLoS One. 2013 Oct 8;8(10):e76837. doi: 10.1371/journal.pone.0076837 (PMC3792877; doi:10.1371/journal.pone.0076837)
Supplement: Table S4 — Calculated binding energy (in Kcal/mol) of BH3Bak with Bcl-xl at different time window. E Bcl-xl + Bak is the energy of complex in membrane averaged over the particular window of time in the independent trajectory no. 1. E Bcl-xl and EBak are energies of respective molecules in water averaged over last 50-100 ns simulation. (DOC) [file pone.0076837.s034.doc]

**Binding energy of BH3^bak^ with Bcl-xl at different time window in trajectory 1 in membrane**

| **Window of time(ns)** | **E _Bcl-xl + BH3_** ^bak^ | **E _Bcl-xl_** | **E_BH3_** ^bak^ | **∆E_Binding_** |
| --- | --- | --- | --- | --- |
| 0-10 | -5196.51 | -4459.28 | -798.64 | 61.41 |
| 10-20 | -5271.09 |  |  | -13.17 |
| 20-30 | -5302.71 |  |  | -44.79 |
| 30-40 | -5311.62 |  |  | -53.7 |
| 40-50 | -5323.17 |  |  | -65.25 |
| 50-60 | -5323.46 |  |  | -65.54 |
| 60-70 | -5329.79 |  |  | -71.87 |
| 70-80 | -5331.73 |  |  | -73.81 |
| 80-90 | -5329.73 |  |  | -71.81 |
| 90-100 | -5326.56 |  |  | -68.64 |
