# Supplementary material for: Dynamics of Bcl-xL in Water and Membrane: Molecular Simulations
Source: PLoS One. 2013 Oct 8;8(10):e76837. doi: 10.1371/journal.pone.0076837 (PMC3792877; doi:10.1371/journal.pone.0076837)
Supplement: Table S5 — Calculated binding energy (in Kcal/mol) of BH3Bak with Bcl-xl at different time window. E Bcl-xl + Bak is the energy of complex in membrane averaged over the particular window of time in the independent trajectory no. 2. E Bcl-xl and EBak are energies of respective molecules in water averaged over last 50-100 ns simulation. (DOC) [file pone.0076837.s035.doc]

**Binding energy of BH3^bak^ with Bcl-xl at different time window in trajectory 2 in membrane**

| **Window of time(ns)** | **E _Bcl-xl + BH3_** ^bak^ | **E _Bcl-xl_** | **E_BH3_** ^bak^ | **∆E_Binding_** |
| --- | --- | --- | --- | --- |
| 0-10 | -5192.73 | -4459.28 | -798.64 | 65.19 |
| 10-20 | -5280.33 |  |  | -22.41 |
| 20-30 | -5292.1 |  |  | -34.18 |
| 30-40 | -5309.96 |  |  | -52.04 |
| 40-50 | -5304.64 |  |  | -46.72 |
| 50-60 | -5304.76 |  |  | -46.84 |
| 60-70 | -5305.01 |  |  | -47.09 |
| 70-80 | -5307.47 |  |  | -49.55 |
| 80-90 | -5310.9 |  |  | -52.98 |
| 90-100 | -5312.34 |  |  | -54.42 |
