# Supplementary material for: Dynamics of Bcl-xL in Water and Membrane: Molecular Simulations
Source: PLoS One. 2013 Oct 8;8(10):e76837. doi: 10.1371/journal.pone.0076837 (PMC3792877; doi:10.1371/journal.pone.0076837)
Supplement: Table S6 — List of trajectories simulated using explicit water and explicit membrane environment. (DOC) [file pone.0076837.s036.doc]

**Details of explicit trajectories**

|  | **System** | | **Medium** | **Number of independent trajectories** | **Length of each independent trajectory** | **Total sampling time** |
| --- | --- | --- | --- | --- | --- | --- |
| 1 | Uncomplexed  Bcl-xl | | TIP3P water | 5 | 10 ns | 50 ns |
|  |  |  | DOPC lipid bilayer | 5 | 10 ns | 50 ns |
| 2 | Bcl-xl+BH3 ^Bak^ | | TIP3P water | 5 | 10 ns | 50 ns |
|  |  |  | DOPC lipid bilayer | 5 | 10 ns | 50 ns |
|  |  | |  |  | Total | 200 ns |
|  | |  | | | | |
